# An overlooked subset of *Cx3cr1*^*wt/wt*^ microglia in the *Cx3cr1*^*CreER-Eyfp/wt*^ mouse has a repopulation advantage over *Cx3cr1*^*CreER-Eyfp/wt*^ microglia following microglial depletion

**DOI:** 10.1186/s12974-022-02381-6

**Published:** 2022-01-21

**Authors:** Kai Zhou, Jinming Han, Harald Lund, Nageswara Rao Boggavarapu, Volker M Lauschke, Shinobu Goto, Huaitao Cheng, Yuyu Wang, Asuka Tachi, Cuicui Xie, Keying Zhu, Ying Sun, Ahmed M. Osman, Dong Liang, Wei Han, Kristina Gemzell-Danielsson, Christer Betsholtz, Xing-Mei Zhang, Changlian Zhu, Martin Enge, Bertrand Joseph, Robert A. Harris, Klas Blomgren

**Affiliations:** 1 Henan Neurodevelopment Engineering Research Center for Children, Children’s Hospital Affiliated to Zhengzhou University, Zhengzhou, China; 2 Department of Women’s and Children’s Health, Karolinska Institutet, Stockholm, Sweden; 3 Applied Immunology and Immunotherapy, Department of Clinical Neuroscience, Karolinska Institutet, Center for Molecular Medicine, Karolinska University Hospital, Stockholm, Sweden; 4 Department of Physiology and Pharmacology, Karolinska Institutet, Stockholm, Sweden; 5 Department of Obstetrics and Gynecology, Nagoya City University Hospital, Nagoya, Japan; 6 Department of Oncology-Pathology, Karolinska Institutet, Stockholm, Sweden; 7 Department of Immunology, Genetics and Pathology, Rudbeck Laboratory, Uppsala University, Uppsala, Sweden; 8 WHO-Centre, Karolinska University Hospital, Stockholm, Sweden; 9 Department of Medicine Huddinge, Karolinska Institutet, Campus Flemingsberg, Huddinge, Sweden; 10 Centre for Brain Repair and Rehabilitation, Institute of Neuroscience and Physiology, University of Gothenburg, Gothenburg, Sweden; 11 Henan Key Laboratory of Child Brain Injury and Henan Pediatric Clinical Research Center, The Third Affiliated Hospital and Institute of Neuroscience, Zhengzhou University, Zhengzhou, China; 12 Institute of Environmental Medicine, Karolinska Institutet, Stockholm, Sweden; 13 Pediatric Oncology, Karolinska University Hospital, Stockholm, Sweden; 14 Neuroimmunology Center, Department of Neurology, Xuanwu Hospital, Capital Medical University, National Center for Neurological Disorders, Beijing, China; 15 Dr Margarete Fischer-Bosch Institute of Clinical Pharmacology, Stuttgart, Germany; 16 University of Tuebingen, Tuebingen, Germany; 17 Department of Obstetrics and Gynecology, Nagoya University Graduate School of Medicine, Nagoya, Japan

**Keywords:** Microglia, Cre, GFP, YFP, *Cx3cr1*, Microglial depletion, Microglial repopulation, Diphtheria toxin subunit A (DTA), Loss of heterozygosity (LOH), Homologous recombination

## Abstract

**Background:**

Fluorescent reporter labeling and promoter-driven Cre-recombinant technologies have facilitated cellular investigations of physiological and pathological processes, including the widespread use of the *Cx3cr1*^*CreER-Eyfp/wt*^ mouse strain for studies of microglia.

**Methods:**

Immunohistochemistry, Flow Cytometry, RNA sequencing and whole-genome sequencing were used to identify the subpopulation of microglia in *Cx3cr1*^*CreER-Eyfp*/*wt*^ mouse brains. Genetically mediated microglia depletion using *Cx3cr1*^*CreER-Eyfp/wt*^*Rosa26*^*DTA/wt*^ mice and CSF1 receptor inhibitor PLX3397 were used to deplete microglia. Primary microglia proliferation and migration assay were used for in vitro studies.

**Results:**

We unexpectedly identified a subpopulation of microglia devoid of genetic modification, exhibiting higher *Cx3cr1* and CX3CR1 expression than *Cx3cr1*^*CreER-Eyfp/wt*^*Cre*^+^*Eyfp*^+^ microglia in *Cx3cr1*^*CreER-Eyfp*/*wt*^ mouse brains, thus termed *Cx3cr1*^high^*Cre*^−^*Eyfp*^−^ microglia. This subpopulation constituted less than 1% of all microglia under homeostatic conditions, but after Cre-driven DTA-mediated microglial depletion, *Cx3cr1*^high^*Cre*^**−**^*Eyfp*^−^ microglia escaped depletion and proliferated extensively, eventually occupying one-third of the total microglial pool. We further demonstrated that the *Cx3cr1*^high^*Cre*^**−**^*Eyfp*^−^ microglia had lost their genetic heterozygosity and become homozygous for wild-type *Cx3cr1*. Therefore, *Cx3cr1*^high^*Cre*^**−**^*Eyfp*^−^ microglia are *Cx3cr1*^*wt/wt*^*Cre*^**−**^*Eyfp*^−^. Finally, we demonstrated that CX3CL1–CX3CR1 signaling regulates microglial repopulation both in vivo and in vitro.

**Conclusions:**

Our results raise a cautionary note regarding the use of *Cx3cr1*^*CreER-Eyfp/wt*^ mouse strains, particularly when interpreting the results of fate mapping, and microglial depletion and repopulation studies.

**Supplementary Information:**

The online version contains supplementary material available at 10.1186/s12974-022-02381-6.

## Background

Microglia are derived from the yolk sac during early embryonic development and represent approximately 10% of the healthy adult brain's total cell population [1, 2]. They play critical roles in maintaining brain development and function [3, 4]. In their homeostatic state, they display a highly ramified morphology, efficiently surveying the central nervous system (CNS) microenvironment, recognizing and clearing cell debris [5]. Microglia play pivotal roles in diverse CNS diseases including neurodevelopmental disorders, neurodegenerative disorders, and high-grade glioma [6–13]. Targeting microglia has thus emerged as an attractive strategy to modulate neuroinflammation in the context of various CNS diseases [14, 15].

The *Cx3cr1* gene is mainly expressed in microglia in the CNS parenchyma, and the CX3CL1/CX3CR1 axis plays a crucial role in microglia–neuron communication [16, 17]. *Cx3cr1* is widely used to genetically label microglia by inserting *Gfp* [18, 19] since this enables microglia tracing, visualization and sorting [1, 20–23]. *Cx3cr1*^*Cre/wt*^ and *Cx3cr1*^*CreER-Eyfp/wt*^ mouse strains are potent, commonly used tools for studies of microglial fate mapping [1, 24], microglial depletion [25–28] and modification of the microglial genome by leveraging floxed target genes [29–31]. These methods have greatly increased our understanding of microglia in CNS homeostasis and disease conditions.

In this study, we report the existence of an unexpected, small population of *Cx3cr1*^high^*Cre*^−^*Eyfp*^−^ microglia in *Cx3cr1*^*CreER-Eyfp/wt*^ mice. After genetically mediated microglial depletion using *Cx3cr1*^*CreER-Eyfp/wt*^*Rosa26*^*DTA/wt*^ mice, *Cx3cr1*^high^*Cre*^−^*Eyfp*^−^ microglia escape depletion, display a repopulation advantage and eventually constitute one-third of the total repopulated microglial pool. We further determined that the *Cx3cr1*^high^*Cre*^−^*Eyfp*^−^ microglia are *Cx3cr1*^*wt/wt*^*Cre*^**−**^*Eyfp*^−^. Finally, we demonstrate the vital role of CX3CL1–CX3CR1 signaling in regulating microglial repopulation post-depletion. Not being aware of this population may result in misinterpretation of the results generated since these cells escape detection (not carrying the *Eyfp* or *Gfp*) and cannot be modified (lacking *Cre* expression) as expected.

## Methods

### Mice

Breeding pairs of *Rosa*26^*DTA/DTA*^ and *Cx3cr1*^*CreER-Eyfp/CreER-Eyfp*^ mice were bought from the Jackson Laboratory (Bar Harbor, ME, USA) with stock numbers 009669 and 021160, respectively. Second generation pups with genotypes *Cx3cr1*^*CreER-Eyfp/wt*^*Rosa26*^*wt/wt*^, *Cx3cr1*^*CreER-Eyfp/wt*^*Rosa26*^*DTA/wt*^, *Cx3cr1*^*CreER-Eyfp/CreER-Eyfp*^*Rosa26*^*wt/wt*^ and *Cx3cr1*^*CreER-Eyfp/CreER-Eyfp*^*Rosa26*^*DTA/wt*^ were used for all experiments. *Cx3cr1*^*Gfp/Gfp*^ mice breeding pairs were bought from the Jackson Laboratory (005582, Bar Harbor, ME, USA), C57BL/6 mice were bought from Charles River (Sulzfeld, Germany), and the pups of *Cx3cr1*^*Gfp/Gfp*^ and C57BL/6 mice were used for the experiments. All mice were housed with a 12:12 h light–dark cycle and had free access to food and water.

### Tam injections

Tamoxifen (T5648-1G, Sigma-Aldrich, Merck, Germany) was dissolved in corn oil (C8267, Sigma-Aldrich, Merck, Germany), and 125 mg/kg or 62.5 mg/kg was injected i.p. every 24 h for 3 (postnatal days 18, 19 and 20) or 10 consecutive days (postnatal days 18–27).

### PLX3397 chow treatment

PLX3397 (HY-16749, MedChemExpress) was formulated with standard chow at 290 mg/kg by SAFE Nutrition Service, France. Four-week-old mice were treated with PLX3397 chow for 3 weeks and sacrificed at different timepoints afterwards.

### Genotyping

DNA was extracted from ear punches and microglia. The following primer sets were used for the genotyping, *Cx3cr1*^*CreER-Eyfp*^: common 5′-AAG ACT CAC GTG GAC CTG CT-3′, Wild-type reverse 5′-AGG ATG TTG ACT TCC GAG TTG-3′, Mutant reverse 5′-CGG TTA TTC AAC TTG CAC CA-3′, Long targeting primer 5′-TGTCCGTATAGGTTGGGAAA-3′; *DTA*: common 5′-AAA GTC GCT CTG AGT TGT TAT-3′, Wild-type reverse 5′-GGA GCG GGA GAA ATG GAT ATG-3′, Mutant reverse 5′-GCG AAG AGT TTG TCC TCA ACC-3′.

### Immunohistochemistry, cell counting, and image analysis

After perfusion with cold PBS, the brain was dissected, immersed in 4% paraformaldehyde (PFA) for 2 days, followed by immersion in 30% sucrose in 0.1 M phosphate buffer until fully saturated. Brain tissues were sagittally sliced into 25 µm sections and stored in tissue cryoprotectant solution (25% ethylene glycol and 25% glycerin in 0.1 M phosphate buffer). The sections were rinsed in Tris-buffered saline (TBS, 50 mM Tris–HCl in 150 mM NaCl, pH 7.5) and then blocked with 3% donkey serum in TBS with 0.1% Triton X-100. After blocking, the sections were incubated overnight in primary antibody solution (Goat anti-Iba-1, 1:500, ab5076, Abcam; Chicken anti-GFP, 1:800, ab13970 Abcam; Rabbit anti-Tmem119, 1:500, ab209064, Abcam; Rabbit anti-P2ry12, 1:500, generated at Harvard Medical School; Rat anti-Ki67, 1:500, 14-5698-82, eBioscience). After washing, the sections were incubated for 2 h at room temperature with a secondary antibody (Donkey anti-goat, 1:1,000, Alexa Fluor 633, 20127, BIOTIUM; Donkey anti-chicken, 1:1000, Alexa Fluor 488, 703-545-155, Jakson Immuno Research; Donkey anti-rabbit, 1:1000, Alexa Fluor 555, 1945911, Invitrogen; Donkey anti-Rat, 1:1,000, Alexa Fluor 555, ab150154, Abcam) together with Hoechst (1:1000, 33342 Life Technologies). The sections were mounted onto glass slides with ProLong® Gold antifade reagent (1925239, Invitrogen). For Iba-1^+^ counting, the sections were blocked with 3% donkey serum in TBS with 0.1% Triton X-100, and then incubated in Rabbit anti-Iba-1 1:1,000 (Wako) overnight. After that, sections were incubated with the secondary antibody (donkey anti-rabbit 1:500, 20215, BIOTIUM) for 2 h, and then endogenous peroxidase activity was blocked with 3% H_2_O_2_ for 10 min. Visualization was performed using Vectastain ABC Elite with 3,3-diaminobenzidine.

Iba-1^+^ microglia were counted using an Axio Imager M2 microscope with Apotome attachment (Carl Zeiss). Cells were counted exhaustively on day 1, day 3, day 7 and day 10 after the final Tam injection. While a fractionator was used to estimate the positive cells on day 42 after the final Tam injection, the counting frame width and height are 150 μm, sample grid X and Y are 300 μm. Thus, all the data has a 2nd estimated coefficient of errors (Schmitz-Hof) less than 0.1 [32]. Then, relative microglia number ratios were calculated with reference to the control group.

All the fluorescence images were captured using an LSM700 laser scanning confocal microscope (Axio-observer Z1; CarlZeiss microscopy, Germany) and analyzed using ZEN software (the black edition; Zeiss).

### Morphological analysis

Confocal microscopy was used to acquire images at 3 µm intervals, with a 40× objective lens used for *Cx3cr1* depletion analysis (Fig. 2g and Additional file 1: Fig. S4G) and a 20× lens used for *Cx3cr1*^*CreER-Eyfp/wt*^ physiological analysis (Additional file 1: Fig. S5A) (Plan-Apochromat lens, 20×/0.8 and 40×/1.3 Oil objective, Carl Zeiss). Microglial morphology was analyzed with the skeleton analysis method using FIJI open-source image analysis software (Image J 1.51s, NIH) [33, 34]. Briefly, the maximum intensity projections of z-series stacks were created. The Iba-1^+^ channel and the CX3CR1-EYFP channel were enhanced in brightness to visualize all processes, followed by de-speckling to eliminate single-pixel backgrounds. Then, each channel image was processed into a binary image, and a topological skeleton image was created (Additional file 1: Fig. S2A). The Analyze Skeleton plugin feature was applied to the whole image frame, and the process length and the number of endpoints were normalized by the cell number per frame. Three morphological parameters were used: (1) total length of the processes, (2) number of endpoints, and (3) microglial process area. The process length and the number of endpoints of CX3CR1^+/+^EYFP^−^Iba-1^+^ cells were calculated by subtracting the CX3CR1-EYFP channel data from the Iba-1 channel data. Each cell's process area was represented as the convex hull area by connecting the process ends using the polygon tool (Additional file 1: Fig. S2B, C) [35]. The selection criteria for the process area analysis were relatively isolated from the processes of the surrounding cells, and whole processes were not truncated and are within the image.

### Microglial isolation

Single-cell suspensions were prepared using a Neural Tissue Dissociation Kit (T, Miltenyi Biotec), and myelin was removed using 38% Percoll. After passing through a 40 μm cell strainer, cells were incubated with the CD11b magnetic bead for 20 min. CD11b^+^ cells were isolated and collected using a magnetic field and MS column (Miltenyi Biotec, Germany).

### Flow cytometry

After microglia isolation, cells were transferred into 96-well V-bottom plates and incubated at 4 °C for 20 min with the following antibodies: yellow dead cell stain kit (L34959, Thermo Fisher Scientific), Near-IR dead cell stain kit (L34975, Thermo Fisher Scientific), CD11b (PerCP-Cyanine5.5, Clone M1/70, BioLegend), CD45 (PE-Cyanine7, Clone 30F11, BioLegend), Ly6C (PE, Clone HK1.4, BioLegend), Ly-6G (V450, Clone 1A8, BD Biosciences), F4/80 (APC, Clone BM8, BioLegend), MHCII (Alexa Fluor 700, Clone M5/114.15.2, BioLegend), Cx3cr1 (Alexa Fluor 700, Clone SA011F11, Biolegend), Cre Recombinase (PE, Clone D7L7L, Cell Signaling Technology) and Ki67 (V450, Clone B56, BD). Cells were run on a Gallios flow cytometer (Beckman Coulter), and the data were analyzed with Kaluza software (Beckman Coulter).

### Cell sorting and bulk RNA sequencing

Cells were sorted into CD11b^+^CD45^+^Ly6C^−^CX3CR1^+^EYFP^+^ and CD11b^+^CD45^+^Ly6C^−^CX3CR1^+^EYFP^−^ populations using a BD Influx Cell Sorter. Briefly, 100 cells were collected directly into lysis buffer, and the library was built by the smart-seq method. Gene expression data were analyzed using Qlucore Omics Explorer 3.4. For two-group and multi-group comparisons of candidate genes, expression was considered as significantly different if *p* < 0.05 using a heteroscedastic two-tailed Student’s *t* test or an *F* test, respectively. For transcriptome-wide analyses, the Benjamini–Hochberg method was used to correct multiple tests.

### Whole-genome sequencing

Whole-genome sequencing was performed essentially as in DNTR-seq [36] with minor modifications. Cell nuclei (1,500–5,000 nuclei per sample) were treated with 15 μl proteinase K (0.4 ml/ml) for 2 h at 50 °C followed by heat inactivation at 80 °C for 30 min. 2 μl of the solution was taken for tagmentation with 1 μl Tn5 stock solution and 1.6 μl reaction buffer (50 mM TAPS, 25 mM MgCl2, 40% PEG 8k; final concentration 8% PEG, 5 mM MgCl2, 10 mM TAPS) and 3.4 ml H2O (final volume 8 μl), incubated at 55 °C for 10 min, and then inactivated by adding 2 μl 0.2% SDS and incubating at 55 °C for 10 min. For barcoding PCR, each sample was split into 4 individual reaction, each barcoded with a unique molecular barcode (fastq files for the replicate reactions were merged after sequencing). 1 μl of the tagmentated DNA solution was used as the template for each barcoding PCR reaction. The PCR program was 72 °C/3 min, 95 °C/30 s, [95 °C/15 s, 67 °C/30 s, 72 °C/1 min] × 15 cycles, 72 °C/5 min, and then 10 °C hold. After PCR amplification, the reactions were cleaned-up by SPRI-beads (at 0.9× volume) for size selection and were pooled together according in equal parts according to their DNA concentration (qubit dsDNA quantification assays, ThermoFisher). The pooled library was then cleaned-up one more time by SPRI-beads (at 0.9× volume) and sequenced on an Illumina NextSeq 550 using the high output 75 bp kit.

Genomic reads were trimmed for adaptor sequences using TrimGalore and mapped to human genome reference build hg38 with the non-genomic parts of the Cx3cr1-CreERT2 targeting vector added as a separate template. Duplicates were removed using Picard MarkDuplicate, along with read pairs with MAPQ < 20. Reads mapping to the part of the vector which is inserted into the genome (position 1173–6936), and to the region surrounding the insertion site (chr9:120,040,000–120,080,000) were extracted from the bam files. Physical coverage (eg. the number of times a base is spanned by paired read mapping positions) was visualized using a custom R script.

### Primary microglia proliferation and migration assay

Primary microglia were cultured as follows: cerebrum tissues were collected from postnatal days 3 mice and used for generating mixed glia cultures in T75 flasks. After 2 weeks of primary culture (DMEM F-12 with 20 ng/ml M-CSF), the microglia were isolated with CD11b magnetic bead sorting (MACS, Miltenyi Biotec) according to the manufacturer’s protocol. For proliferation assay, isolated CD11b positive cells were seeded at a density of 0.2 × 10^5^ on the coverslips (83.1840.002, Sarstedt) in 24-well plate for 2 days with DMEM F-12 medium containing 10% FCS and 20 ng/ml M-CSF, followed by replacement with serum-free medium (DMEM F-12) and serum-free medium (DMEM F-12) with 100 ng/ml CX3CL1 (472-FF-025/CF, R&D systems). 24 h later the cells were fixed by 4% paraformaldehyde, then incubated overnight in primary antibody solution (Goat anti-Iba-1, 1:500, ab5076, Abcam; Rat anti-Ki67, 1:500, 14-5698-82, eBioscience). After washing, the coverslips were incubated for 2 h at room temperature with a secondary antibody (Donkey anti-goat, 1:1000, Alexa Fluor 488, 1942238, invitrogen; Donkey anti-Rat, 1:1000, Alexa Fluor 555, ab150154, Abcam). The rate of ki67 positive cells in Iba-1 positive cells were calculated as the proliferating rate of microglia. For migration assay, isolated CD11b positive cells were seeded at a density of 0.5 × 10^5^ in an Incucyte ImageLock 96-well plate (4379, Sartorius) for overnight with the addition of 10% FCS and 20 ng/ml M-CSF. On the following day, a standardized scratched wound was induced in each well simultaneously using an Incucyte WoundMaker kit (Cat. No. 4493, Sartorius), followed by replacement with serum-free, M-CSF free DMEM/F-12 medium containing indicated concentration of CX3CL1 (472-FF-025/CF, R&D systems). The plate was then placed in an Incucyte Zoom System, and live images were taken every 2 h. The images were analyzed according to the manual of Incucyte Zoom.

## Results

### Identification of a subset of *Cx3cr1*^high^*Cre*^−^*Eyfp*^−^ parenchymal microglia in *Cx3cr1*^*CreER-Eyfp/wt*^ mice

**Fig. 1:**
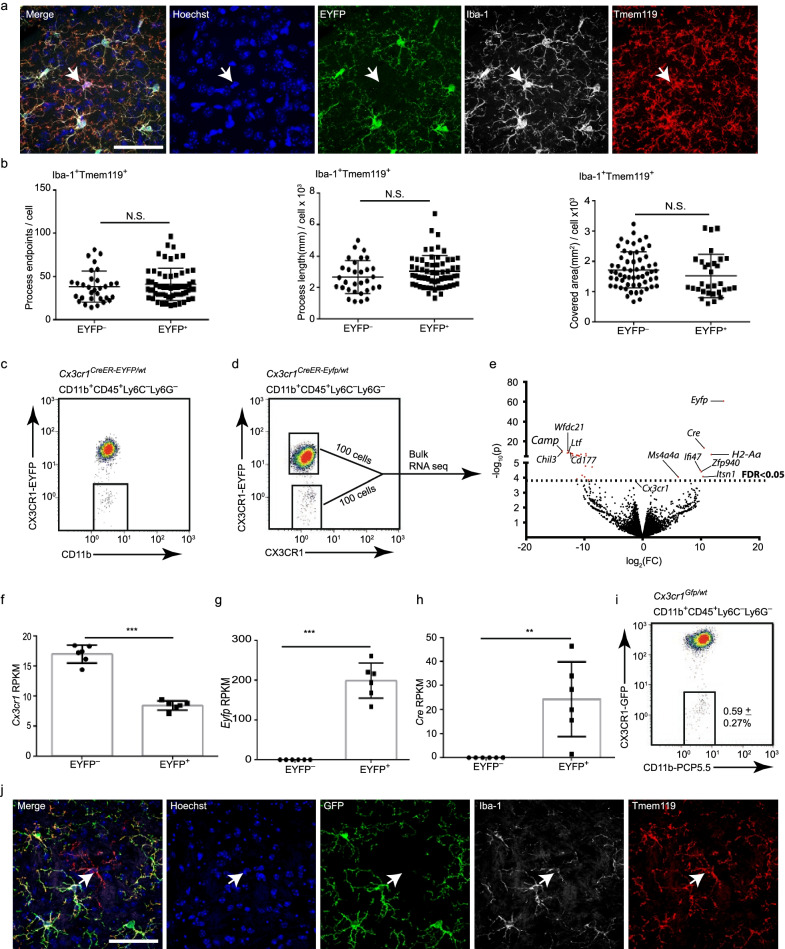
*Cx3cr1*^high^*Cre*^−^*Eyfp*^−^ microglia in *Cx3cr1*^*CreER-Eyfp/wt*^ mice. **a** Representative images of Hoechst, EYFP, Iba-1 and Tmem119 immunohistochemical staining in the brains of *Cx3cr1*^*CreER-Eyfp/wt*^ mice. White arrows point to the EYFP^−^Iba-1^+^Tmem119^+^ cells. Scale bar, 50 μm. **b** Comparison of morphology between EYFP^+^Iba-1^+^ microglia and EYFP^−^Iba-1^+^ microglia in *Cx3cr1*^*CreER-Eyfp/wt*^ mice. The graph shows process endpoints (left, *p* = 0.0773), process length (middle, *p* = 0.6047), and process occupied area (right, *p* = 0.1253) by Student’s two-tailed unpaired *t* test. *n* = 59 for EYFP^+^Iba-1^+^ cells and *n* = 31 for EYFP^−^Iba-1^+^ cells from four *Cx3cr1*^*CreER-Eyfp/wt*^ mice, mean ± s.d. **C** Representative dot plots of EYFP^−^ microglia in the brains of *Cx3cr1*^*CreER-Eyfp/wt*^ mice (gated as CD11b^+^CD45^+^Ly6C^−^ly6G^−^). **d** Representative dot plots of expression of EYFP and CX3CR1, *n* = 8. **e** Volcano plot showing the DEGs with FDR < 0.05, the indicated genes are *Cx3cr1*, all upregulated genes and the top 5 down-regulated genes. **f** The reads per kilobase of transcript, per million mapped reads (RPKM) of *Cx3cr1* between EYFP^−^ and EYFP^+^ microglia from the RNA-seq data, *p* < 0.001 by paired Student’s two-tailed *t* test. **g**, **h** The RPKM of *EYFP* and *Cre* between EYFP^−^ and EYFP^+^ microglia from the RNA-seq data, **p < 0.01, ***p < 0.001 by paired Student’s two-tailed *t* test. **I** Dot plots of flow cytometry analysis of *Cx3cr1*^*GFP/wt*^ mice, values in plots are the ratio of GFP^−^ microglia to total microglia, *n* = 6 mice, mean ± s.d. **j** Representative images of GFP, Iba-1 and Tmem119 triple staining in *Cx3cr1*^*GFP/wt*^ mice, the arrows indicating cells expressing Hoechst, Iba-1, Tmem119, but not GFP, *n* = 6 mice. Scale bar, 50 μm

In *Cx3cr1*^*CreER-Eyfp/wt*^ mice, the protein-coding exon of one of the *Cx3cr1* alleles is replaced by the *Cre-ERT2* fusion gene and the enhanced yellow fluorescent protein gene (*Eyfp*), implying that all CX3CR1^+^ cells (mainly microglia in the CNS parenchyma) should express both Cre-ERT2 and EYFP. Unexpectedly, we observed a subpopulation of Iba-1^+^Tmem119^+^EYFP^**−**^ cells with microglia-like morphology in the CNS parenchyma of *Cx3cr1*^*CreER-Eyfp/wt*^ mice under homeostatic conditions (Fig. 1a and Additional file 1: Fig. S1a). These Iba-1^+^Tmem119^+^EYFP^**−**^ cells and Iba-1^+^Tmem119^+^EYFP^**+**^ microglia exhibited similar morphologies as measured by process complexity (numbers of endpoints, length) and area occupied (Fig. 1b and Additional file 1: Fig. S2). Flow cytometric analysis (gating strategy depicted in Additional file 1: Fig. S3a) confirmed the existence of EYFP^−^ cells in *Cx3cr1*^*CreER-Eyfp/wt*^ mice, accounting for less than 1% of total CD11b+CD45+ Ly6C–Ly6G– cells at 3–15 weeks, with no significant differences between ages. One outlier sample in 9-week age mice was excluded from the analysis, having 6% of EYFP^−^ cells of the total CD11b^+^CD45^+^ Ly6C^−^Ly6G^−^ pool (Fig. 1c and Additional file 1: Fig. S3b). Furthermore, we observed that both EYFP^+^ microglia and EYFP^−^ microglia-like cells were CX3CR1^+^ (Fig. 1d). These data indicate that CX3CR1^+^EYFP^−^ cells are resident microglia.

To further confirm this finding, we performed fluorescence-activated cell (FAC) sorting of CX3CR1^+^EYFP^+^ and CX3CR1^+^EYFP^−^ cells (gated as CD11b^+^CD45^+^Ly6C^−^Ly6G^−^CX3CR1^+^) followed by RNA sequencing (Fig. 1d). To investigate the *Cre* and *Eyfp* expression, the RNA sequencing reads were mapped on the mouse genome integrated with the *CreERT2-Eyfp* knockin-allele. We determined that both CX3CR1^+^EYFP^+^ and CX3CR1^+^EYFP^−^ cells expressed similar levels of specific markers for myeloid cells (*Hexb*, *CD68*, *Fcgr1*, *Adgre1*, *Itgam*, *Sparc*, *C1qa*, *Aif1*, and *Fcer1g*) and microglia (*P2ry12*, *Tmem119*, and *Sall1*) (data not included), further confirming that both CX3CR1^+^EYFP^−^ and CX3CR1^+^EYFP^+^ cells were CNS parenchymal microglia. Next, the transcriptomes of CX3CR1^+^EYFP^−^ and CX3CR1^+^EYFP^+^ microglia were compared, indicating 32 differently expressed genes (DEGs) with a false discovery rate (FDR) < 0.05 (Additional file 2: Table S1). The top 5 downregulated genes in CX3CR1^+^EYFP^+^ microglia were *Wfdc21*, *Camp*, *Chil3*, *Ltf*, and *Cd177*, while all the upregulated genes in CX3CR1^+^EYFP^+^ microglia were *Eyfp*, *Cre*, *H2-Aa*, *Ifi47*, *Zfp940*, *Ms4a4a*, and *Itsn1* (Fig. 1e). Furthermore, we determined that CX3CR1^+^EYFP^−^ microglia expressed twice as much *Cx3cr1* mRNA compared with CX3CR1^+^EYFP^+^ microglia (Fig. 1f). Moreover, CX3CR1^+^EYFP^−^ microglia did not express *Eyfp* or *Cre* mRNA (Fig. 1g, h). We thus defined that CX3CR1^+^EYFP^−^ microglia are *Cx3cr1*^high^*Cre*^−^*Eyfp*^−^.

To assess the variation of *Cx3cr1* expression in C57BL/6 mice, single-cell RNA sequencing data from our previous publication [37] were reanalyzed in CX3CR1^+^ microglia from the hippocampus. No clusters with high or low *Cx3cr1* expression were detected (Additional file 1: Fig. S3c). The *Cx3cr1* expression was normally distributed in CX3CR1^+^ microglia (Additional file 1: Fig. S3d), supporting that the difference in *Cx3cr1* expression levels between *Cx3cr1*^high^*Cre*^−^*Eyfp*^−^ and *Cx3cr1*^*CreER-Eyfp/wt*^*Cre*^+^*Eyfp*^+^ microglia in *Cx3cr1*^*CreER-Eyfp/wt*^ mice was attributed to the genetic modification.

To confirm that *Cx3cr1*^high^*Cre*^−^*Eyfp*^−^microglia were not an anomaly of one specific mouse strain, we investigated the microglial populations in *Cx3cr1*^*GFP/wt*^ mice (JAX, 005582) by flow cytometry (gating strategy depicted in Additional file 1: Fig. S3a) and immunohistochemistry. The results revealed that Iba-1^+^Tmem119^+^GFP^−^ cells with microglial morphology could also be detected in the *Cx3cr1*^*GFP/wt*^ adult mouse brain, constituting 0.59% ± 0.27% of total microglia (Fig. 1i, j).

### *Cx3cr1*^high^*Cre*^−^*Eyfp*^−^ microglia repopulated and became a dominant subgroup of the repopulated microglia pool following microglial depletion

**Fig. 2:**
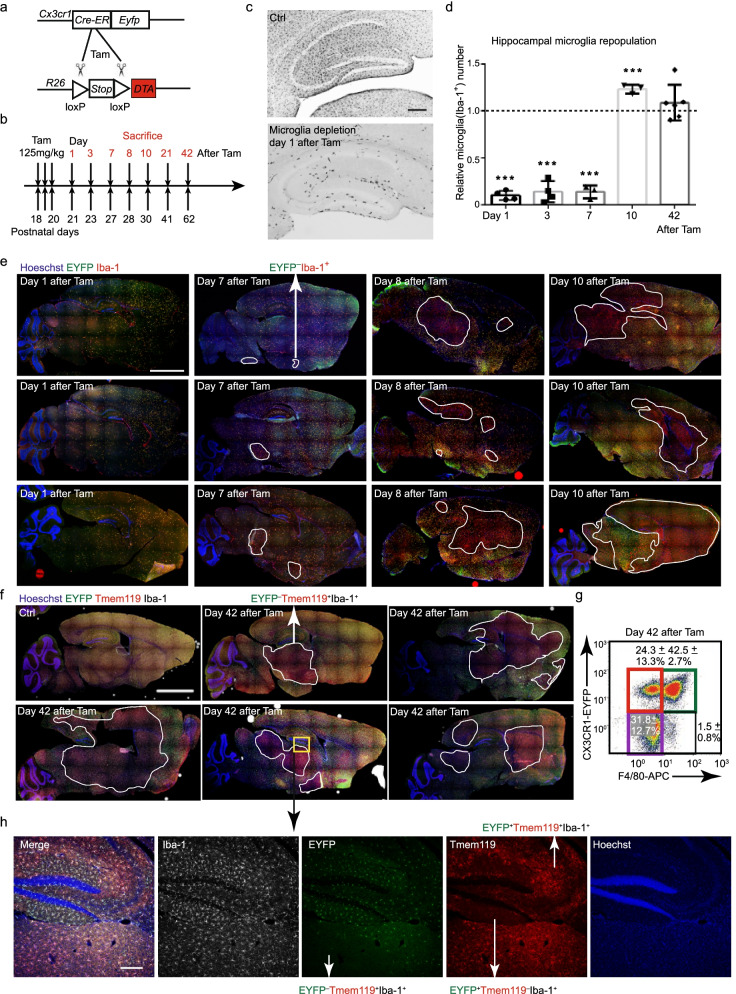
*Cx3cr1*^high^*Cre*^−^*Eyfp*^−^ microglia were repopulated following microglial depletion. **a** Schematic figure of *Cx3cr1*^*CreER-Eyfp/wt*^*Rosa26*^*DTA/wt*^ mice following Tam injections. **b** Tam was injected on 3 consecutive days at postnatal 18, 19, and 20 days. The mice were then sacrificed for IHC, flow cytometry, and morphological analysis at days 1, 3, 7, 8, 10, 21, 42 after the final Tam injection. **c** Representative images of Iba-1 staining in the hippocampus of *Cx3cr1*^*CreER-Eyfp/wt*^ mice (Ctrl group) and *Cx3cr1*^*CreER-Eyfp/wt*^*Rosa26*^*DTA/wt*^ mice (depletion group) at day 1 after the final Tam injection. Scale bar, 200 μm. **d** Relative numbers of Iba-1^+^ microglia compared with Ctrl *Cx3cr1*^*CreER-Eyfp/wt*^ mice. *n* = 3–4, mean ± s.d. *p < 0.05, ***p < 0.01 by Student’s two-tailed unpaired *t* test. **e** EYFP and Iba-1 staining of *Cx3cr1*^*CreER-Eyfp/wt*^*Rosa26*^*DTA/wt*^ mice at days 1, 7, 8, 10 after the final Tam injection. The white curve depicts the distribution of EYFP– microglia in *Cx3cr1*^*CreER-Eyfp/wt*^*Rosa26*^*DTA/wt*^ mice. EYFP^−^ microglia are located within the enclosed areas, *n* = 3 per timepoint. Scale bar, 2 mm. **f** Representative EYFP, Iba-1, Tmem119 triple staining of *Cx3cr1*^*CreER-Eyfp/wt*^ (Ctrl group) and *Cx3cr1*^*CreER-Eyfp/wt*^*Rosa26*^*DTA/wt*^ (depletion group) mice. The white curve depicted the distribution of EYFP^−^ microglia in*Cx3cr1*^*CreER-Eyfp/wt*^*Rosa26*^*DTA/wt*^ mice. EYFP^−^ microglia are located within the enclosed areas, *n* = 5 mice per group. Scale bar, 2 mm. **g** Representative dot plots depicting 4 different subpopulations of newly repopulated microglia (EYFP^+^ F4/80^low^, EYFP^−^ F4/80^low^, EYFP^+^ F4/80^hi^, EYFP^−^ F4/80^hi^) at day 42 after the final Tam injection. **h** High magnification of EYFP, Iba-1, Tmem119 triple staining images showing Tmem119^+^EYFP^−^, Tmem119^+^EYFP^+^, and Tmem119^−^EYFP^+^ microglial distribution. Scale bar, 200 μm

To investigate whether *Cx3cr1*^high^*Cre*^−^*Eyfp*^−^ microglia are capable of filling the vacant microglial niche following microglial depletion, we first crossed *Cx3cr1*^*CreER-Eyfp/CreER-Eyfp*^ and *Rosa*26^*DTA/DTA*^ mouse strains (Fig. 2a, JAX, 009669) to breed *Cx3cr1*^*CreER-Eyfp/wt*^*Rosa26*^*DTA/wt*^ mice. Following tamoxifen (Tam) injections, *Cx3cr1*^*CreER-Eyfp/wt*^ cells in the brain parenchyma (mainly *Cx3cr1*^*CreER-Eyfp/wt*^*Cre*^+^*Eyfp*^+^ microglia) can be depleted through the intracellular release of diphtheria toxin [38]. To track dynamic microglial alterations in the brain, *Cx3cr1*^*CreER-Eyfp/wt*^* Rosa26*^*DTA/wt*^ and *Cx3cr1*^*CreER-Eyfp/wt*^ mice were sacrificed at different timepoints (days 1, 3, 7, 8, 10, 21, and 42) after three consecutive Tam injections on postnatal days 18, 19, and 20, respectively (Fig. 2b). Iba-1^+^ microglia were quantified by immunohistochemistry, revealing that approximately 89.7 ± 2.4%, 90.9 ± 0.3%, and 94.5 ± 1.4% of the microglia were depleted by the first day after the final Tam injection in the hippocampus, cortex, and cerebellum, respectively, (Fig. 2c, d, Additional file 1: Fig. S4a, b). Moreover, newly repopulated microglia were noted 1 week later, amounting to 21.9 ± 8.5% in the cortex and 34.9 ± 5.9% in the cerebellum compared to control *Cx3cr1*^*CreER-Eyfp/wt*^ mice (Additional file 1: Fig. S4a, b). We also recorded an overabundance of microglia 10 days after the final Tam injection, 123.3 ± 2.7% in the hippocampus and 139.8 ± 18.6% in the cerebellum compared to the control group (Fig. 2d and Additional file 1: Fig. S4a). No significant differences were observed in the numbers of newly repopulated microglia 42 days after the final Tam injection, compared with the baseline level (Fig. 2c, d, Additional file 1: Fig. S4a, b).

The numbers of *Cx3cr1*^high^*Cre*^−^*Eyfp*^−^ (EYFP^−^Iba1^+^) microglia increased from day 1 to day 3 after the final Tam injection (Additional file 1: Fig. S4c, d). Distinct clusters of *Cx3cr1*^high^*Cre*^−^*Eyfp*^−^ microglia were formed by day 7, further expanded on days 8 and 10, and then maintained 42 days after the final Tam injection (Fig. 2e, f). Interestingly, newly repopulated *Cx3cr1*^high^*Cre*^−^*Eyfp*^−^ microglial clusters were distributed in a stochastic manner with a considerable regional variation between different mice, not co-localizing with *Cx3cr1*^*CreER-Eyfp/wt*^* Cre*^+^*Eyfp*^+^ microglia clusters (Fig. 2e, f). We have previously demonstrated that high and low expression of F4/80 can be used to distinguish resident repopulated microglia and peripheral infiltrating microglia-like cells following experimental microglial depletion [38]. Flow cytometric analysis revealed 3 main subsets of microglia-like cells after repopulation in *Cx3cr1*^*CreER-Eyfp/wt*^*Rosa26*^*DTA/wt*^ mice (Fig. 2g). These included EYFP^+^F4/80^low^, EYFP^−^F4/80^low^, and EYFP^+^F4/80^hi^, likely representing resident *Cx3cr1*^*CreER-Eyfp/wt*^*Cre*^+^*Eyfp*^+^ microglia, resident *Cx3cr1*^high^*Cre*^−^*Eyfp*^−^ microglia, and peripherally derived microglia-like cells, respectively (Fig. 2g). The finding was further confirmed using Immunohistochemistry (IHC), again demonstrating mainly 3 repopulated microglial subgroups expressing EYFP^+^Tmem119^+^, EYFP^−^Tmem119^+^, and EYFP^+^Tmem119^−^, respectively (Fig. 2h).

**Fig. 3:**
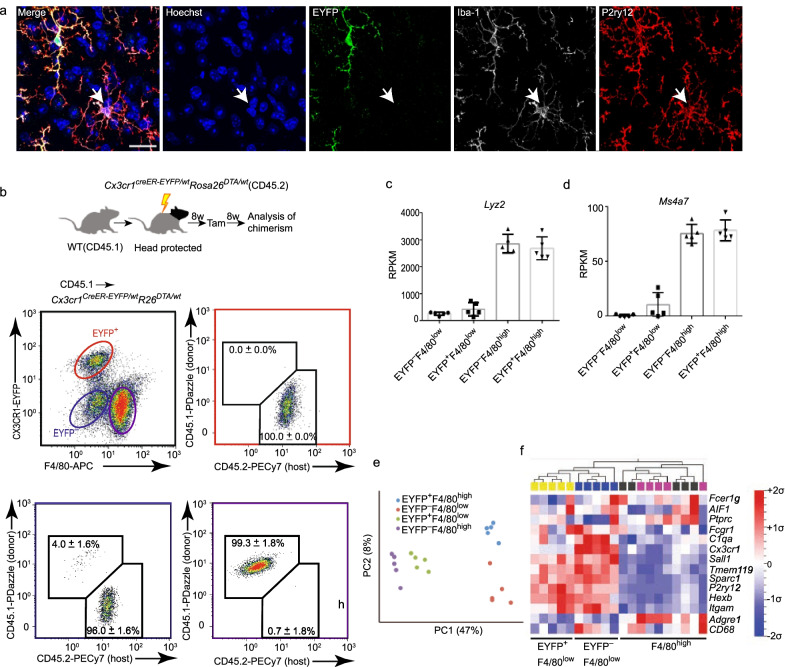
Repopulated EYFP^−^ microglia originate from the resident microglia pool. **a** Representative images of EYFP, Iba-1, P2ry12 triple staining on newly repopulated microglia of *Cx3cr1*^*CreER-Eyfp/wt*^*Rosa26*^*DTA/wt*^ mice. Scale bar, 20 μm. **b** Experimental chimerism setup to determine the origin of repopulated *Cx3cr1*^high^*Cre*^−^*Eyfp*^−^ microglia after microglia depletion. Middle-left panel gated on CD11b^+^CD45^+^Ly6C^−^Ly6G^−^EYFP^−^ microglia. Middle-right panel with red color rectangle gated on F4/80^low^EYFP^+^ microglia. Bottom-left panel with blue color gated on F4/80^low^ EYFP^−^ microglia. Bottom-right with purple color gated on F4/80^high^ cells. *n* = 5–12, mean ± s.d. **c**, **d** Bar graphs show RPKM of *Ms4a7* and *Lyz2*, *n* = 5, mean ± s.d. **e** Principal component analysis of genes *p* < 0.05. **f** Heat map visualization of specific genes on macrophage and microglia. Data is presented as mean-centered sigma-normalized

We next addressed if these newly repopulated *Cx3cr1*^high^*Cre*^−^*Eyfp*^−^ microglia were derived from the periphery. We and others have previously demonstrated that the empty microglial niche can be repopulated within weeks through resident microglia proliferation and concomitant infiltration of monocytes [38, 39]. Two novel specific surface markers (Tmem119 and P2ry12) were used to identify CNS-resident microglia [40, 41]. Our results demonstrated that all newly repopulated *Cx3cr1*^high^*Cre*^−^*Eyfp*^−^ microglia expressed both Tmem119 (Fig. 2h) and P2ry12 (Fig. 3a). Furthermore, the vast majority of *Cx3cr1*^high^*Cre*^−^*Eyfp*^−^ microglia had low expression of F4/80 (Fig. 2g).

To confirm the CNS origin of *Cx3cr1*^high^*Cre*^−^*Eyfp*^−^ microglia, we reanalyzed data from our previous publication [38]. Specifically, *Cx3cr1*^*CreER-Eyfp/wt*^*Rosa26*^*DTA/wt*^ and *Cx3cr1*^*CreER-Eyfp/wt*^ mice (CD45.2) were exposed to head-protected irradiation and transplanted with congenic CD45.1 bone marrow, Tam was injected 8 weeks after reconstitution, and the chimeras were analyzed another 8 weeks later (Fig. 3b). We determined that the vast majority of the F4/80^high^ cells were derived from CD45.1^+^ donor cells, while all *Cx3cr1*^*CreER-Eyfp/wt*^*Cre*^+^*Eyfp*^+^ (EYFP^+^F4/80^low^) microglia originated from CD45.2^+^ residential microglia in both *Cx3cr1*^*CreER-Eyfp/wt*^ and *Cx3cr1*^*CreER-Eyfp/wt*^*Rosa26*^*DTA/wt*^ chimeras, and that the vast majority of the *Cx3cr1*^high^*Cre*^−^*Eyfp*^−^ (EYFP^−^F4/80^low^) microglia were derived from CD45.2^+^ residential microglia, indicating their CNS origin.

This notion was further confirmed by RNA sequencing of the 4 groups of repopulated microglia expressing EYFP^+^F4/80^low^, EYFP^−^F4/80^low^, EYFP^+^F4/80^high^, and EYFP^−^F4/80^high^, respectively (Fig. 2g). Specifically, our results demonstrated that the F4/80^low^ groups had lower expression of signature genes of engrafted macrophages, including *Ms4a7* and *Lyz2*, compared to the F4/80^high^ groups (Fig. 3c, d). Principle component analysis (PCA) indicated that the greatest difference was evident between F4/80^low^ and F4/80^high^ groups (Fig. 3e). We further investigated the expression levels of 14 macrophage- and microglia-related genes [42], and EYFP^+^F4/80^low^ and EYFP^−^F4/80^low^ microglia expressed similar patterns, while EYFP^+^F4/80^high^ and EYFP^−^F4/80^high^ microglia expressed similar patterns (Fig. 3f). For example, the F4/80^high^ groups expressed higher levels of *Adgre1* (F4/80) and *Ptprc* (CD45) than did the F4/80^low^ groups (Fig. 3f), supporting the efficiency of our sorting strategy and the peripheral source of the F4/80^high^ groups. F4/80^high^ and F4/80^low^ groups expressed similar levels of *Fcer1g*, *Aif1* (Iba-1), and *Cd68*. Moreover, F4/80^high^ groups expressed lower levels of *Fcgr1*, *C1qa*, *Cx3cr1*, *Sall1*, *Tmem119*, *sparc1*, *P2ry12*, *Hexb* and *Itgam* (CD11b) (Fig. 3f). Taken together, newly repopulated *Cx3cr1*^high^*Cre*^−^*Eyfp*^−^microglia originate from the resident *Cx3cr1*^high^*Cre*^−^*Eyfp*^−^ microglia pool.

Collectively, although only less than 1% *Cx3cr1*^high^*Cre*^−^*Eyfp*^−^ microglia exist in the *Cx3cr1*^*CreER-Eyfp/wt*^ mouse brain, they can account for one-third of the total newly repopulated microglia pool following conditional genetic depletion in *Cx3cr1*^*CreER-Eyfp/wt*^*Rosa26*^*DTA/wt*^ mice.

### *Cx3cr1*^high^*Cre*^−^*Eyfp*^−^ microglia have lost the *CreERT2-Eyfp* fusion gene and reveal a wild-type *Cx3cr1* genotype

We next investigated the newly repopulated CNS-derived (F4/80^low^) microglia. Consistent with our findings in naive mice, EYFP^−^ microglia sorted after repopulation lacked *Cre* and *Eyfp* expression but expressed twice the levels of *Cx3cr1* compared to EYFP^+^ microglia (Additional file 1: Fig. S5a–c). Moreover, flow cytometric analysis indicated a higher expression of CX3CR1 protein in *Cx3cr1*^high^*Cre*^−^*Eyfp*^−^ (EYFP^−^) microglia compared to *Cx3cr1*^*CreER-Eyfp/wt*^*Cre*^+^*Eyfp*^+^ (EYFP^+^) microglia (Additional file 1: Fig. S5d, e).

**Fig. 4:**
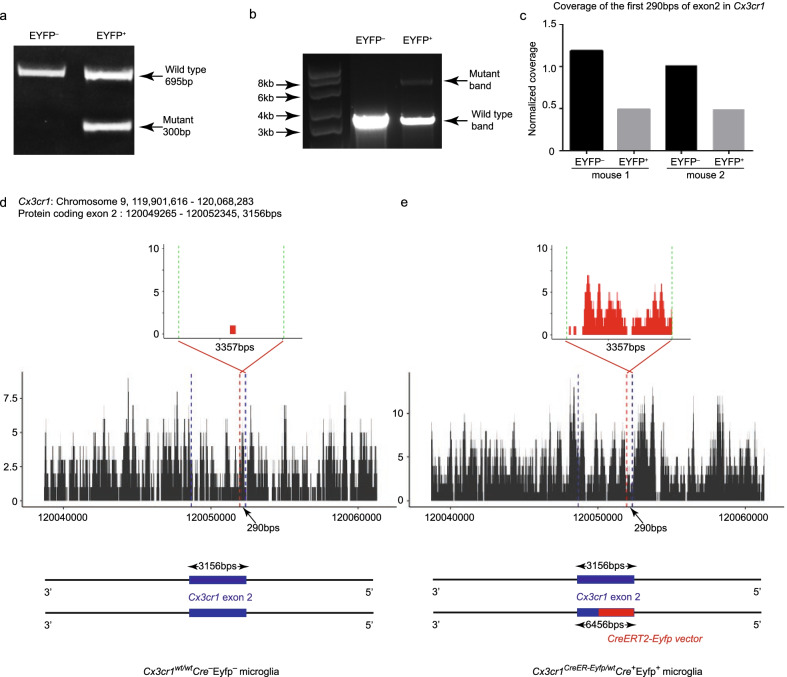
*Cx3cr1*^high^*Cre*^−^*Eyfp*^−^ microglia are *Cx3cr1*^*wt/wt*^*Cre*^−^*Eyfp*^−^. **a**, **b** DNA electrophoresis of PCR product from EYFP^−^ and EYFP^+^ microglia. **c** Normalized coverage of the first 290 bps in exon 2 of *Cx3cr1* in both *Cx3cr1*^high^*Cre*^−^*Eyfp*^−^ and *Cx3cr1*^*CreER-Eyfp/wt*^*Cre*^+^*Eyfp*^+^ microglia, indicating the integrity of the protein-coding exon 2 of *Cx3cr1*, mouse 1 and mouse 2 are two biological replicates. **d**, **e** Whole-genome sequencing of *Cx3cr1*^high^*Cre*^−^*Eyfp*^−^ microglia (**d**) and *Cx3cr1*^*CreER-Eyfp/wt*^*Cre*^+^*Eyfp*^+^ microglia (**e**). The top panel represents the sequencing mapping on the *CreERT2-Eyfp* vector; the middle panel represents the sequencing mapping on chromosome 9; the bottom panel is a schematic graph showing the homozygosity of the exon 2 of *Cx3cr1*

Given the lack of *Cre* and *Eyfp* expression on the mRNA and protein levels, we reasoned that the *CreER-Eyfp* knockin-allele was either epigenetically/genetically silenced or deleted. We thus investigated *Cx3cr1*^high^*Cre*^−^*Eyfp*^−^ microglia at the DNA level. Genotyping of DNA extracted from *Cx3cr1*^high^*Cre*^−^*Eyfp*^−^ and *Cx3cr1*^*CreER-Eyfp/wt*^*Cre*^+^*Eyfp*^+^ microglia revealed that *Cx3cr1*^high^*Cre*^−^*Eyfp*^−^ microglia apparently lacked the *CreERT2-Eyfp* insert (Fig. 4a). This analysis was performed using the primers and protocols Jax lab recommended, which only target part of the *CreERT2-Eyfp* knockin-allele (Additional file 1: Fig. S6a). We, therefore, further designed primers and optimized protocols to target longer sequences, including the whole *Cx3cr1* exon 2 containing the inserted *CreERT2-Eyfp* fusion gene (Additional file 1: Fig. S6a). The mutant band was not detected in *Cx3cr1*^high^*Cre*^−^*Eyfp*^−^ microglia (Fig. 4b), again indicating that they lack the *CreERT2-Eyfp* insert. Whole-genome sequencing of the DNA extracted from *Cx3cr1*^high^*Cre*^−^*Eyfp*^−^ and *Cx3cr1*^*CreER-Eyfp/wt*^*Cre*^+^*Eyfp*^+^ microglia further confirmed the complete absence of the *CreERT2-Eyfp* knockin-allele in *Cx3cr1*^high^*Cre*^−^*Eyfp*^−^ microglia. Interestingly, the coverage of the integrated *Cx3cr1* coding exon in *Cx3cr1*^high^*Cre*^−^*Eyfp*^−^ microglia is twice that of the coverage in the *Cx3cr1*^*CreER-Eyfp/wt*^*Cre*^+^*Eyfp*^+^ microglia (Fig. 4c–e, Additional file 1: Fig. S6b, e), indicating that *Cx3cr1*^high^*Cre*^−^*Eyfp*^−^ microglia are *Cx3cr1*^*wt/wt*^*Cre*^−^*Eyfp*^−^.

We next addressed the origin of *Cx3cr1*^*wt/wt*^*Cre*^−^*Eyfp*^−^ microglia. The experimental *Cx3cr1*^*CreER-Eyfp/wt*^*Rosa26*^*DTA/wt*^ mice were bred from male *Cx3cr1*^*CreER-Eyfp/CreER-Eyfp*^*Rosa*26^*DTA/DTA*^ and female *Cx3cr1*^*wt/wtp*^*Rosa*26^*wt/wt*^ mice. We first excluded the possibility of maternally derived microglia since the *Cx3cr1*^*wt/wt*^*Cre*^−^*Eyfp*^−^ microglia inherited the paternal *DTA* knockin-allele (Additional file 1: Fig. S6d). We then hypothesized that homologous recombination during microglial mitosis leads to loss of the *CreERT2-Eyfp* insert. To address this hypothesis, we tested whether breeding *Cx3cr1*^*CreER-Eyfp/CreER-Eyfp*^*Rosa*26^*DTA/wt*^ mice would result in the loss of the *CreERT2-Eyfp* insert, given the fact that there is no wild type allele to recombine with. Our results revealed that the EYFP^−^F4/80^low^ microglia, here denoted as *Cx3cr1*^*wt/wt*^*Cre*^−^*Eyfp*^−^ microglia, were not detected in *Cx3cr1*^*CreER-Eyfp/CreER-Eyfp*^*Rosa26*^*DTA/wt*^ mice after genetically microglial depletion and repopulation (Additional file 1: Fig. S6e). These results indicate that homologous recombination is a possible mechanism leading to loss of heterozygosity (LOH) of *Cx3cr1*^*wt/wt*^*Cre*^−^*Eyfp*^−^ microglia in *Cx3cr1*^*CreER-Eyfp/wt*^ mice.

### *CX3CL1*–*CX3CR1 signaling regulates resident microglia repopulation*

**Fig. 5:**
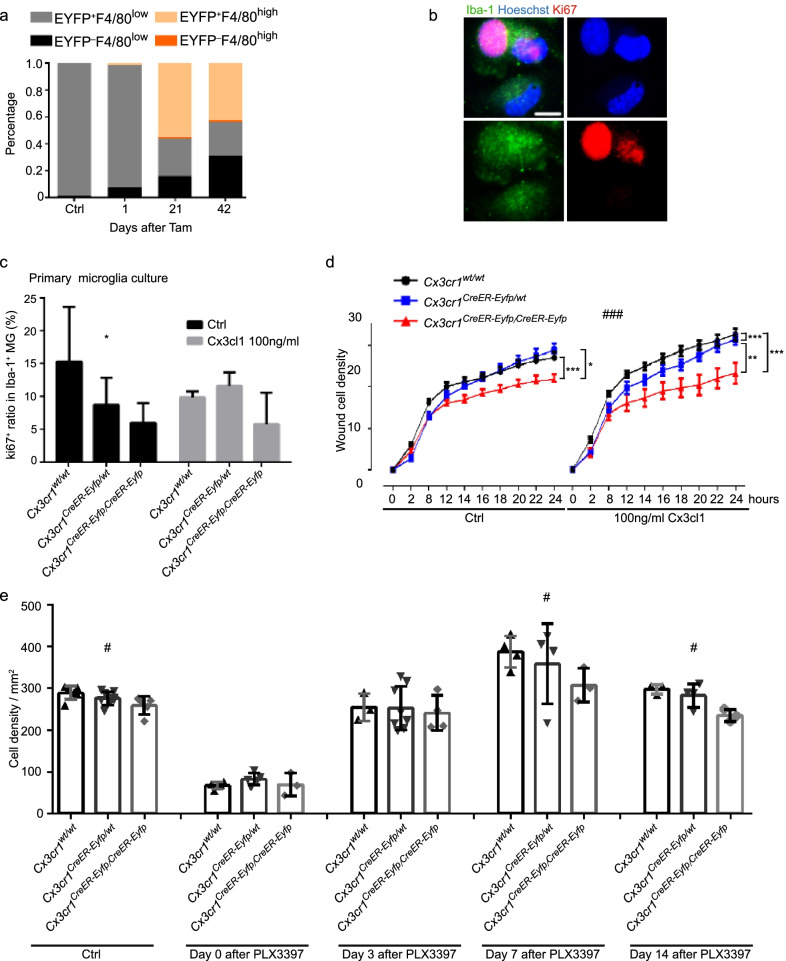
Competitive advantage of higher CX3CR1 expressed microglia during microglial repopulation. **a** Different percentages of EYFP^+^ F4/80^low^, EYFP^−^ F4/80^low^, EYFP^+^ F4/80^hi^, and EYFP^−^ F4/80^hi^ microglia in total microglia at Ctrl, day 1, 21 and 42 after Tam injections. **b** Representative images of Hoechst, Iba-1 and Ki67 staining in the primary cultured microglial. **c** Bar graph showing the rate of ki67^+^ cells in Iba-1^+^ cells in *Cx3cr1*^*wt/wt*^, *Cx3cr1*^*CreER-Eyfp/wt*^, *Cx3cr1*^*CreER-Eyfp/ CreER-Eyfp*^ primary microglia, and the rate of ki67^+^ cells in Iba-1^+^ cells in *Cx3cr1*^*wt/wt*^, *Cx3cr1*^*CreER-Eyfp/wt*^, and *Cx3cr1*^*CreER-Eyfp/CreER-Eyfp*^ primary microglia after adding 100 ng/ml CX3CL1 in the medium. *n* = 3–4, mean ± s.d. *p < 0.05 by two-way ANOVA of factor *Cx3cr1*, no interactions were between factors of *Cx3cr1* and CX3CL1. **d** Migration assay: the left panel showing would density in *Cx3cr1*^*wt/wt*^, *Cx3cr1*^*CreER-Eyfp/wt*^, *Cx3cr1*^*CreER-Eyfp/CreER-Eyfp*^ primary microglia culture. *n* = 6, mean ± s.d. *p < 0.05, ***p < 0.001 by one-way repeated ANOVA; the right panel showing would density in *Cx3cr1*^*wt/wt*^, *Cx3cr1*^*CreER-Eyfp/wt*^, *Cx3cr1*^*CreER-Eyfp/CreER-Eyfp*^ primary microglia culture after adding 100 ng/ml CX3CL1. *n* = 6, mean ± s.d. **p < 0.01, ***p < 0.001 by one-way repeated ANOVA; before and after adding CX3CL1 were compared, ^###^*p* < 0.001 by paired *t* test. **e** Graph showing cell density in the whole brain of control group, day 0, day 3 day 7 and day 14 after ceasing PLX3397 chow treatment in *Cx3cr1*^*wt/wt*^, *Cx3cr1*^*CreER-Eyfp/wt*^, *Cx3cr1*^*CreER-Eyfp/CreER-Eyfp*^ mice, *n* = 3–8, mean ± s.d. ^#^*p* < 0.05 by One-way ANOVA

We investigated whether the *Cx3cr1*^*wt/wt*^*Cre*^−^*Eyfp*^−^ microglia have a competitive advantage during repopulation following depletion due to their higher expression of *Cx3cr1*, analogous to what was reported in a study of the retina [26]. Our results revealed that after depletion, the *Cx3cr1*^*wt/wt*^*Cre*^−^*Eyfp*^−^ (EYFP^−^F4/80^low^) microglia indeed had an advantage over *Cx3cr1*^*CreER-Eyfp/wt*^*Cre*^+^*Eyfp*^+^ (EYFP^+^F4/80^low^) microglia during repopulation. Their numbers increased more quickly, reaching similar numbers as the *Cx3cr1*^*CreER-Eyfp/wt*^*Cre*^+^*Eyfp*^+^ microglia despite originating from a smaller number of surviving cells (Fig. 5a). To investigate whether this repopulation advantage is due to higher *Cx3cr1* expression, their proliferative capacity was assayed in *Cx3cr1*^*wt/wt*^, *Cx3cr1*^*CreER-Eyfp/wt*^, and *Cx3cr1*^*CreER-Eyfp/CreER-Eyfp*^ primary culture microglia. We observed that microglial proliferation rates decreased dose-dependently with decreasing CX3CR1 expression, but independently of the presence of the ligand, CX3CL1 (Fig. 5b, c). Furthermore, a scratch assay revealed that microglial migration was decreased in *Cx3cr1*^*CreER-Eyfp/CreER-Eyfp*^ microglia compared to *Cx3cr1*^*wt/wt*^ and *Cx3cr1*^*CreER-Eyfp/wt*^ microglia. After adding exogenous CX3CL1, microglial migration rates increased in *Cx3cr1*^*wt/wt*^ and *Cx3cr1*^*CreER-Eyfp/wt*^ microglia, more so in *Cx3cr1*^*wt/wt*^ than *Cx3cr1*^*CreER-Eyfp/wt*^, but not in *Cx3cr1*^*CreER-Eyfp/CreER-Eyfp*^ microglia (Fig. 5d). These data indicate that the CX3XL1/CX3CR1 axis is important in microglial repopulation following depletion.

To further confirm the role of CX3CR1 in regulating microglial repopulation. We depleted microglia using PLX3397 chow in *Cx3cr1*^*wt/wt*^, *Cx3cr1*^*CreER-Eyfp/wt*^, and *Cx3cr1*^*CreER-Eyfp/CreER-Eyfp*^ mice. Previous studies have demonstrated that blood-borne infiltrating cells do not contribute to microglia repopulation following microglial depletion using PLX3397 administered in chow [43]. Approximately 70% of the microglia were depleted after 21 days PLX3397 treatment and there was no difference in depletion efficiency between the 3 genotypes. Microglia repopulated quickly 3 days after ceasing treatment, without any differences between the 3 genotypes. However, 7 days after repopulation the *Cx3cr1*^*wt/wt*^ group exhibited more microglia than did the *Cx3cr1*^*CreER-Eyfp/CreER-Eyfp*^ group, reaching 20% higher numbers than the control group, while the *Cx3cr1*^*CreER-Eyfp/CreER-Eyfp*^ group stopped at the control, pre-depletion level and the *Cx3cr1*^*CreER-Eyfp/wt*^ group displayed intermediate numbers (Fig. 5e), further indicating that CX3CR1 plays a role in repopulation when the niche is filling up.

Collectively, our results show that *Cx3cr1*^high^*Cre*^−^*Eyfp*^−^ microglia are *Cx3cr1*^*wt/wt*^*Cre*^−^*Eyfp*^−^. They have an intrinsic, competitive repopulation advantage, which is at least partly due to higher *Cx3cr1* expression than in *Cx3cr1*^*CreER-Eyfp/wt*^ microglia.

## Discussion

The *Cx3cr1*^*CreER-Eyfp*^ mouse line constitutes a central tool for studying microglia biology [30]. In the present study, we unexpectedly identified the presence of *Cx3cr1*^*wt/wt*^*Cre*^−^*Eyfp*^−^ microglia lacking *CreERT2*-*Eyfp* locus in the *Cx3cr1*^*CreER-Eyfp/wt*^ mouse brain, which can be repopulated after microglial depletion following Tam injections in *Cx3cr1*^*CreER-Eyfp/wt*^*Rosa26*^*DTA/wt*^ mice.

One previous study reported that all repopulated microglia were derived from CX3CR1^+^ cells, as measured by fate mapping in *Cx3cr1*^*CreER::Ai14*^ mice. All CX3CR1^+^ microglia were tdTomato^+^, and all Iba-1^+^ cells were tdTomato^+^, leading to the interpretation that all the repopulated Iba-1^+^ cells arose from the surviving CX3CR1^+^ cells following microglial depletion [43]. However, in both our *Cx3cr1*^*CreER-Eyfp/wt*^ and *Cx3cr1*^*GFP/wt*^ transgenic mice, the presence of *Cre*^−^EYFP^−^ and GFP^−^ microglia was recorded. Proliferating GFP^−^ microglia in *Cx3cr1*^GFP/+^ mice have also been reported following microglia depletion using the CSF1 receptor inhibitor PLX3397 [18]. In that study, the authors showed that proliferating GFP^−^ cells could become Iba-1^+^ microglia; however, whether these Iba-1^+^GFP^−^ microglia were also CX3CR1^+^ was not addressed [18]. Our results indicated that these cells could be de facto microglia expressing all thus far established canonical markers and that they existed before microglial depletion and repopulation. Another study reported that most newly repopulated microglia were tdTomato^+^ using the CSF1 receptor inhibitor PLX5622 in *Cx3cr1*^*CreER/*+*:tdTomato*^ mice, and the Iba1^+^tdTomato^−^ cells were interpreted as being infiltrating peripheral monocytes [26]. However, based on our study, we consider that the Iba1^+^tdTomato^−^ cells could be derived from the local microglial cell pool, which does not carry the transgene *Cx3cr1*^*CreER*^. Furthermore, another study demonstrated that EYFP^−^ microglia escaped Cre-mediated recombination and could repopulate the CNS following microglial depletion in *Rosa26*^−*STOP−Eyfp*^*Cx3cr1*^*CreER*^:iDTR mice [39]. Phagocytosis and activation of microglia is, at least partly, dependent on the CX3CR1/CX3CRL axis [44–49]. Hence, the EYFP–microglia, expressing twice the amount of CX3CR1, might have a different phagocytosis capacity. In light of these findings, interpretation of results from *Cx3cr1*^*CreER/wt*^ and *Cx3cr1*^*GFP/wt*^ transgenic mice should be made with caution.

We consider two possible explanations for the presence of *Cx3cr1*^*wt/wt*^*Cre*^−^*Eyfp*^−^ microglia in the *Cx3cr1*^*CreER-Eyfp/wt*^ mouse brain: (i) maternal-derived macrophages or microglia, or (ii) LOH of microglia during mitosis. The possibility of maternal-derived microglia or macrophages was excluded by our results, as the *Cx3cr1*^*wt/wt*^*Cre*^−^*Eyfp*^−^ microglia inherited the paternal DTA gene. Alternatively, LOH due to homologous recombination could explain the existence of *Cx3cr1*^*wt/wt*^*Cre*^−^*Eyfp*^−^ microglia in the *Cx3cr1*^*CreER-Eyfp/wt*^ mouse brain. LOH is a phenomenon whereby the cells only possess the genetic information from one of the parental chromosomes, as previously described in the cancer cells [50] and mammalian cells in vivo and in vitro [51–53]. Our results showing a similar percentage of *Cx3cr1*^*wt/wt*^*Cre*^−^*Eyfp*^−^ microglia in each of the heterozygous *Cx3cr1*^*CreER-Eyfp/wt*^ mouse brains and the absence of *Cx3cr1*^*wt/wt*^*Cre*^−^*Eyfp*^−^ microglia in the homozygous *Cx3cr1*^*CreER-Eyfp/CreER-Eyfp*^ mouse brain support the theory of LOH through homologous recombination during mitosis. The mechanisms of microglial LOH in *Cx3cr1*^*CreER-Eyfp/wt*^ and *Cx3cr1*^*GFP/wt*^ mouse strains and whether the phenomena of LOH also occurs in other analogous genetic modified heterozygous strains need to be further investigated.

Microglial depletion and repopulation studies have expanded our knowledge of microglia in physiological and pathological states, exerting favorable effects in different preclinical disease models [54, 55]. Moreover, new microglia rapidly repopulate the brain parenchyma following microglial depletion, although the origin of these newly repopulated microglia has been debated. Elmore and colleagues reported that CX3CR1^+^GFP^−^ cells were potential microglial precursor cells during the microglial repopulation period. However, this viewpoint has been challenged, and newly repopulated microglia are proposed to only arise from the surviving microglia [56]. This is further supported using fate mapping approaches showing that The new forming microglia only temporary expression nestin, and no microglia were derived from None microglia nestin^+^ cells [43]. Our RNA sequencing data revealed no increased expression of precursor of stem cell genes in *Cx3cr1*^*wt/wt*^*Cre*^−^*Eyfp*^−^ microglia. Moreover, microglial single-cell data from wild type mice revealed no high *Cx3cr1-*expressing microglia that clustered separately from other microglia. Rather, the cells expressing higher levels of Cx3cr1 were evenly distributed (Additional file 1: Fig. S3C, D). Taken together, these data support that *Cx3cr1*^*wt/wt*^*Cre*^−^*Eyfp*^−^ microglia are not precursors or stem cells. How these surviving microglia could survive depletion and whether they were unaffected by the depletion period remain unclear. Microglia cannot actually be fully depleted using currently available depletion models [54] and the existence of a microglial subset that may be resistant to depletion has been previously proposed [57]. *Cx3cr1*^*wt/wt*^*Cre*^−^*Eyfp*^−^ microglia could be one such subset, at least in the *Cx3cr1*-*Cre* derived depletion setting. Our results indicate that both unaffected *Cx3cr1*^*wt/wt*^*Cre*^−^*Eyfp*^−^ microglia and surviving *Cx3cr1*^*CreER-Eyfp/wt*^*Cre*^+^*Eyfp*^+^ microglia repopulate the brain competitively, with contribution from peripherally derived macrophages. Moreover, it is widely known that individual microglia occupy non-overlapping spatial territories [58, 59]. Herein, we report a novel finding that non-overlapping niches exist between repopulated *Cx3cr1*^*wt/wt*^*Cre*^−^*Eyfp*^−^ and *Cx3cr1*^*CreER-Eyfp/wt*^*Cre*^+^*Eyfp*^+^ microglia; however, related signal pathways need to be further investigated.

Tissue macrophages with a self-renewing capacity are seeded during embryonic development, and some macrophages can be replaced or renewed postnatally by peripheral monocytes [60–65]. In the brain, the resident microglial pool is self-renewing without contribution from peripheral monocytes during the whole life span [1]. However, in disease states, such as CNS injury and neurodegenerative diseases, monocytes can infiltrate the brain and become microglia-like cells, albeit with different functionalities [66]. Here we used a mouse model in which peripheral monocytes entered the brain after microglial depletion [38]. Our results demonstrate that the main transcriptomic difference is noted between the F4/80^high^ and F4/80^low^ groups, but not between the EYFP^−^ and EYFP^+^ groups after competitive microglial repopulation, which indicates that the infiltrating monocytes, imprinted by the CNS microenvironment, are different from the resident repopulated microglia, and confirming our previous publication [38].

Microglia replacement therapy has been proposed for CNS diseases linked to microglial dysfunctions or gene mutations. Replacing microglia by genetically modified or engineered cells may hold promise for distinct CNS diseases, yet the factors regulating competitive engraftment of different populations including microglia, monocytes and engineered cells are poorly understood [67, 68]. CX3CR1 regulates microglia colonization and distribution in the brain [69], and *Cx3cr1* gene-deleted mice exhibited lower microglial density in the developing brain [70]. The CX3CL1–CX3CR1 axis also regulates microglial repopulation following microglial depletion in the mouse retina [26], but whether this axis regulates competitive microglial repopulation in the brain has not previously been addressed. Our results indicate that repopulating microglia originate from three different predominant sources, resident microglia, including both *Cx3cr1*^*wt/wt*^*Cre*^−^*Eyfp*^−^ and *Cx3cr1*^*CreER-Eyfp/wt*^ microglia, competing with infiltrating peripheral-derived microglia-like cells. Our data demonstrate that *Cx3cr1*^*wt/wt*^*Cre*^−^*Eyfp*^−^ microglia have a competitive advantage over *Cx3cr1*^*CreER-Eyfp/wt*^ microglia. Furthermore, resident microglia lacking *Cx3cr1* (from *Cx3cr1*^*CreER-Eyfp/CreER-Eyfp*^ mice) were unable to compete with the peripheral-derived microglia-like cells following microglial depletion. This indicates that the resident microglia repopulation, but not peripheral derived microglia-like cell repopulation relies on CX3CR1. The proliferation rate of microglia in vitro was decreased in *Cx3cr1* gene-depleted primary microglia, and this was not affected when the cells were challenged with CX3CL1 protein. This likely explains the repopulation advantage of *Cx3cr1*^*wt/wt*^*Cre*^−^*Eyfp*^−^ over *Cx3cr1*^*CreER-Eyfp/wt*^ microglia post-depletion, at least partly. *Cx3cr1* deficiency decreased the microglial migration rate, consistent with previous studies reporting that *Cx3cr1* deficiency impaired microglia migration in vivo [71, 72] and in vitro [73]. However, microglial migration was not impaired in *Cx3cr1*^*CreER-Eyfp/wt*^ microglia. CX3CL1 increased migration rates of *Cx3cr1*^*wt/wt*^*Cre*^−^*Eyfp*^−^ microglia but not of *Cx3cr1*^*CreER-Eyfp/wt*^ and *Cx3cr1*^*CreER-Eyfp/CreER-Eyfp*^ microglia, which indicates that CX3CL1–CX3CR1 regulates microglial migration. The migration rate is higher in *Cx3cr1*^*wt/wt*^*Cre*^−^*Eyfp*^−^ than *Cx3cr1*^*wt/wt*^*Cre*^−^*Eyfp*^−^ microglia after adding CX3CL1, which further explains the repopulation advantage of *Cx3cr1*^*wt/wt*^*Cre*^−^*Eyfp*^−^ over *Cx3cr1*^*CreER-Eyfp/wt*^ microglia post-depletion. Taken together, we conclude that the CX3CL1–CX3CR1 axis is important for the resident microglial repopulation and for competition with peripheral monocyte-derived microglia-like cells. Thus, limiting residential microglia repopulation by inhibiting CX3CL1–CX3CR1 signaling improves the microglial replacement efficiency by peripheral derived monocytes.

## Conclusions

A small portion (less than 1%) of *Cx3cr1*^*wt/wt*^*Cre*^−^*Eyfp*^−^ microglia do not carry genetic labels in the widely used *Cx3cr1*^*GFP/wt*^ or *Cx3cr1*^*CreER-Eyfp/wt*^ mouse models. Not being aware of this population may lead to significant data misinterpretation since these cells may escape detection (not carrying the *Eyfp* or *Gfp*) and cannot be modified (lacking *Cre* expression) as expected. We further demonstrate the important role of the CX3CL1–CX3CR1 axis in regulation of microglial repopulation post-depletion. These findings raise an important cautionary note, not only when using the strains mentioned above but also for other strains that might display similar phenomena.

## Supplementary Information

**Additional file 1: Figure S1.** Additional data related to Fig. 1. **A** Representative 8 sections of Hoechst staining of *Cx3cr1*^*CreER-Eyfp/wt*^ mouse brain sequential sagittal slices, the numbers representing the location and number of EYFP^−^ microglia in that location, each color represents one mouse, *n* = 6. **Figure S2.** Morphology analysis. **A** The process to prepare topological skeleton from the original photomicrographs. The full-size maximum intensity projection images (top) were processed into binary images (middle) and then skeletonized (bottom) following the ImageJ plugin protocol. Cropped images (right) from the original full-size images (left) were shown to improve the visualization. Scale bar, 50 μm. **B** The Analyze Skeleton plugin was applied, and skeletonized endpoints were tagged purple, the slab is orange, and the junction is pink. The tagged data are summarized as total length (sum of endpoints, slab, and junction) and the total number of endpoints. **C** The process area is represented as the convex hull area by connecting the process ends using the ImageJ polygon tool. **Figure S3.** Additional data related to Fig. 1. **A** Gating strategies of flow cytometry analyses. **B** EYFP^−^ microglia ratio of total microglia at 3 weeks, 4 weeks, 6 weeks, 9 weeks and 15 weeks old mice, *n* = 4, 4, 6, 4, respectively, mean ± s.d. No significant difference by one-way ANOVA. **C** UMAP plots from single-cell sequencing, each dot represents one cell, the color represents the expression levels of *Cx3cr1*. **D** Histogram plot of *Cx3cr1*. The *x*-axis represents *Cx3cr1* expression read counts; the *y*-axis represents cell numbers. **Figure S4.** Additional data related to Fig. 2. **A** Graph showing relative Iba-1^+^ microglia at 1 day, 3 days and 7 days after the final Tam injection, respectively; *n* = 3–4, mean ± s.d. *p < 0.05, **p < 0.01, ***p < 0.001 by Student’s two-tailed unpaired *t* test. **B** Representative images of Iba-1 microglia staining of *Cx3cr1*^*CreER-Eyfp/wt*^ mice and *Cx3cr1*^*CreER-Eyfp/wt*^* Rosa26*^*DTA/wt*^ mice at 1 day after the final Tam injection in cortex and cerebellum. Scale bar, 200 μm. **C** Representative 3 sequential sagittal sections (25 µm/section, with 36 sections interval) after Hoechst staining of *Cx3cr1*^*CreER-Eyfp/wt*^* Rosa26*^*DTA/wt*^ mice brains. The numbers represent the location and number of *Cx3cr1*^high^*Cre*^−^*Eyfp*^−^ microglia at 1 day and 3 days after the final Tam injection, each color represents one mouse, *n* = 3. **D** Quantitative data showing the total number of microglia in the 3 sections in Ctrl group, 1 day and 3 days after the final Tam injection,* n* = 4, 3, 3, mean ± s.d. **Figure S5.** Additional data related to Fig. 4. **A**–**C** Bar graphs showing RPKM of *Cre, EYFP,* and *Cx3cr1*, respectively, *n* = 5, mean ± s.d. ***p < 0.001 by Student’s two-tailed unpaired *t* test. **D** The expression of CX3CR1 in both *Cx3cr1*^*CreER-Eyfp/wt*^*Cre*^+^*Eyfp*^+^ and *Cx3cr1*^high^*Cre*^−^*Eyfp*^−^ microglia in *Cx3cr1*^*CreER-Eyfp/wt*^*Rosa26*^*DTA/wt*^ mice at day 42 after the final Tam injection. **E** Bar graph showing the mean fluorescence intensity (MFI) of CX3CR1, *n* = 4, mean ± s.d. ***p < 0.001 by Student’s two-tailed unpaired *t* test. **Figure S6.** Additional data related to Fig. 4. **A** Schematic diagram showed how the primers were designed. The red color indicates the common primers used in all the PCR reactions; the green and blue color indicate the WT primer and Mutant primer used in PCR reaction for Fig. 4f; the orange color indicates the long PCR product targeting primer used in PCR reaction for Fig. 4g. **B** Whole-genome sequencing of *Cx3cr1*^high^*Cre*^−^*Eyfp*^−^ microglia in mouse 2. The top panel is the sequencing mapping on the *CreERT2-Eyfp* vector; the middle panel is the sequencing mapping on chromosome 9; the bottom panel is the schematic graph showing the homozygosity of the exon 2 of *Cx3cr1*. **C** Whole-genome sequencing of *Cx3cr1*^*CreER-Eyfp/wt*^*Cre*^+^*Eyfp*^+^ microglia. The top panel is the sequencing mapping *CreERT2-Eyfp* vector; the middle panel is the sequencing mapping the chromosome 9; the bottom panel is the schematic graph showing the heterogeneity of the exon 2 of *Cx3cr1*. **D** DNA electrophoresis of PCR product from EYFP^−^ and EYFP^+^ microglia. **E** Representative dot plots showing the percentage of EYFP^+^F4/80^low^, EYFP^−^F4/80^low^, EYFP^+^F4/80^high^, and EYFP^−^F4/80^high^ repopulated microglia at day 42 after Tam in *Cx3cr1*^*CreER-Eyfp/ CreER-Eyfp*^* Rosa26*^*DTA/wt*^ mice, mean ± s.d.

**Additional file 2: Table S1.** Gene list including Gene symbols, Log2 fold changes, P-values and FDR values. The table shows FDR values and P-values in ascending order.

## Data Availability

The data supporting the findings are available upon request from the corresponding author. The RNA and DNA sequencing data generated during the current study are available in the public repository GEO with accession numbers GSE 186700 and PRJNA 778933, respectively.

## Abbreviations

DTA: Diphtheria toxin subunit A
LOH: Loss of heterozygosity
CNS: Central nervous system
*Eyfp*: Enhanced yellow fluorescent protein gene
FAC: Fluorescence-activated cell
Tam: Tamoxifen
