# Supplementary material for: An overlooked subset of Cx3cr1wt/wt microglia in the Cx3cr1CreER-Eyfp/wt mouse has a repopulation advantage over Cx3cr1CreER-Eyfp/wt microglia following microglial depletion
Source: J Neuroinflammation. 2022 Jan 21;19:20. doi: 10.1186/s12974-022-02381-6 (PMC8783445; doi:10.1186/s12974-022-02381-6)
Supplement: Supplementary file 1 — Additional file 1: Figure S1. Additional data related to Fig. 1. A Representative 8 sections of Hoechst staining of Cx3cr1CreER-Eyfp/wt mouse brain sequential sagittal slices, the numbers representing the location and number of EYFP− microglia in that location, each color represents one mouse, n = 6. Figure S2. Morphology analysis. A The process to prepare topological skeleton from the original photomicrographs. The full-size maximum intensity projection images (top) were processed into binary images (middle) and then skeletonized (bottom) following the ImageJ plugin protocol. Cropped images (right) from the original full-size images (left) were shown to improve the visualization. Scale bar, 50 μm. B The Analyze Skeleton plugin was applied, and skeletonized endpoints were tagged purple, the slab is orange, and the junction is pink. The tagged data are summarized as total length (sum of endpoints, slab, and junction) and the total number of endpoints. C The process area is represented as the convex hull area by connecting the process ends using the ImageJ polygon tool. Figure S3. Additional data related to Fig. 1. A Gating strategies of flow cytometry analyses. B EYFP− microglia ratio of total microglia at 3 weeks, 4 weeks, 6 weeks, 9 weeks and 15 weeks old mice, n = 4, 4, 6, 4, respectively, mean ± s.d. No significant difference by one-way ANOVA. C UMAP plots from single-cell sequencing, each dot represents one cell, the color represents the expression levels of Cx3cr1. D Histogram plot of Cx3cr1. The x-axis represents Cx3cr1 expression read counts; the y-axis represents cell numbers. Figure S4. Additional data related to Fig. 2. A Graph showing relative Iba-1+ microglia at 1 day, 3 days and 7 days after the final Tam injection, respectively; n = 3–4, mean ± s.d. *p < 0.05, **p < 0.01, ***p < 0.001 by Student’s two-tailed unpaired t test. B Representative images of Iba-1 microglia staining of Cx3cr1CreER-Eyfp/wt mice and Cx3cr1CreER-Eyfp/wt Rosa26DTA/wt mice at 1 da [file 12974_2022_2381_MOESM1_ESM.pdf]

Fig. S1

a

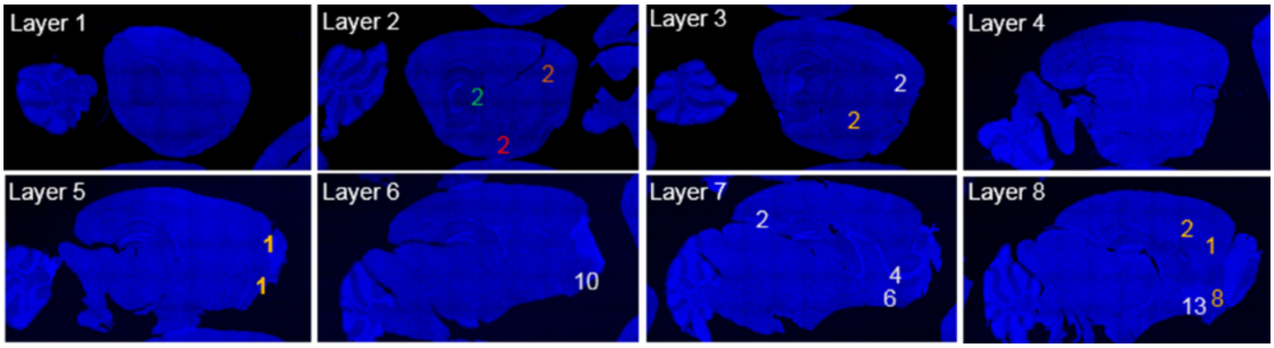

Fig. S2

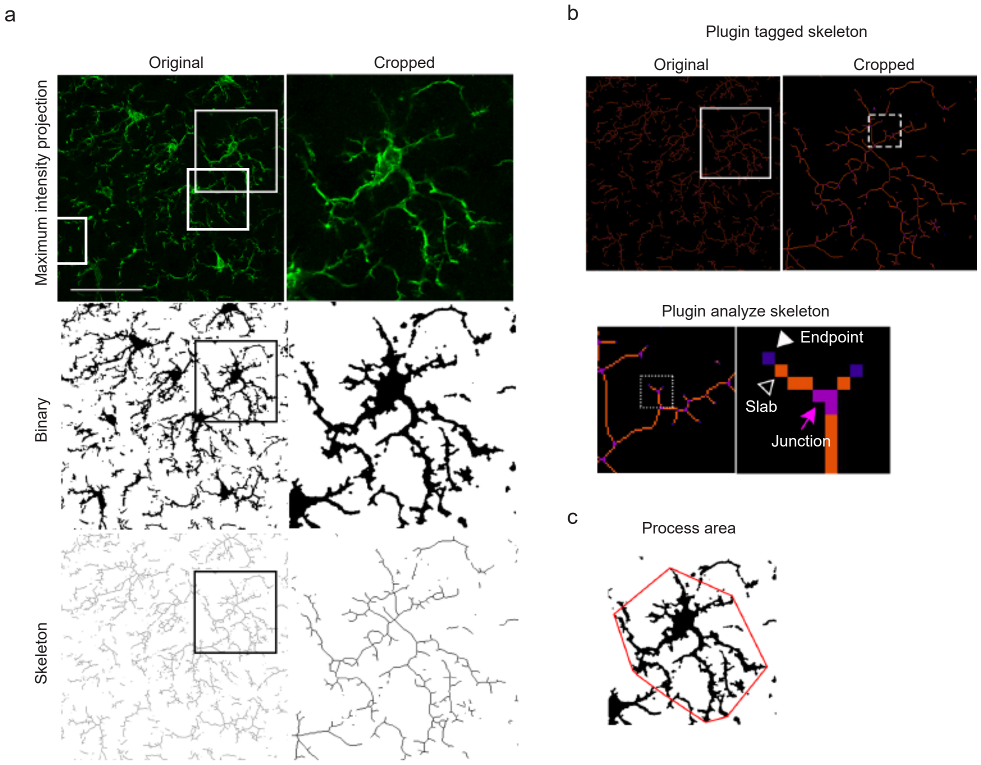

Fig. S3

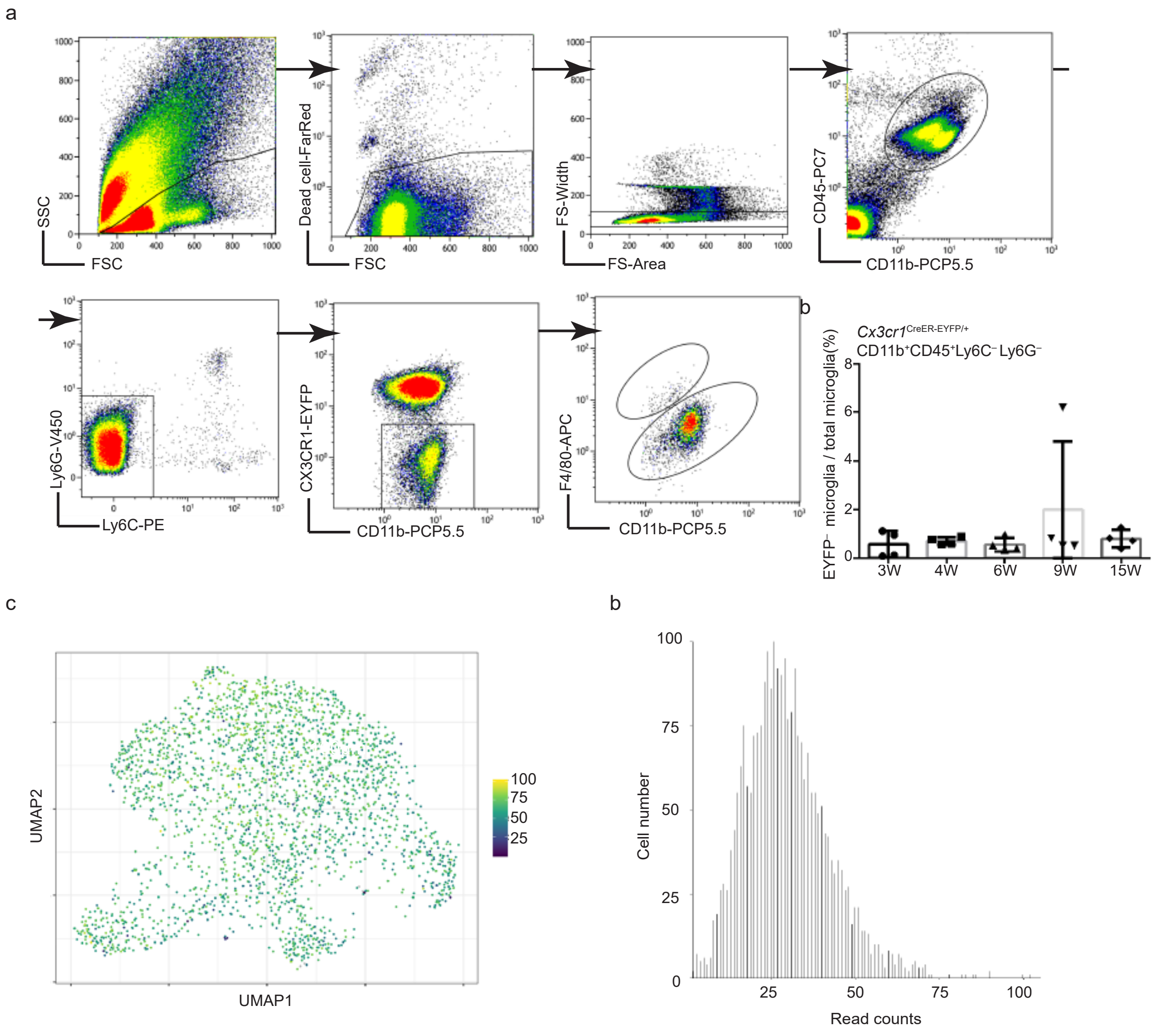

a
SSC
FSC
Dead cell-FarRed
FSC
FS-Width
FS-Area
CD45-PC7
CD11b-PCP5.5
Ly6G-V450
Ly6C-PE
CX3CR1-EYFP
CD11b-PCP5.5
F4/80-APC
CD11b-PCP5.5
b
Cx3cr1CreER-EYFP/+
CD11b+CD45+Ly6C−Ly6G−
EYFP− microglia / total microglia(%)
3W
4W
6W
9W
15W
c
UMAP2
UMAP1
100
75
50
25
b
Cell number
Read counts

Fig. S4

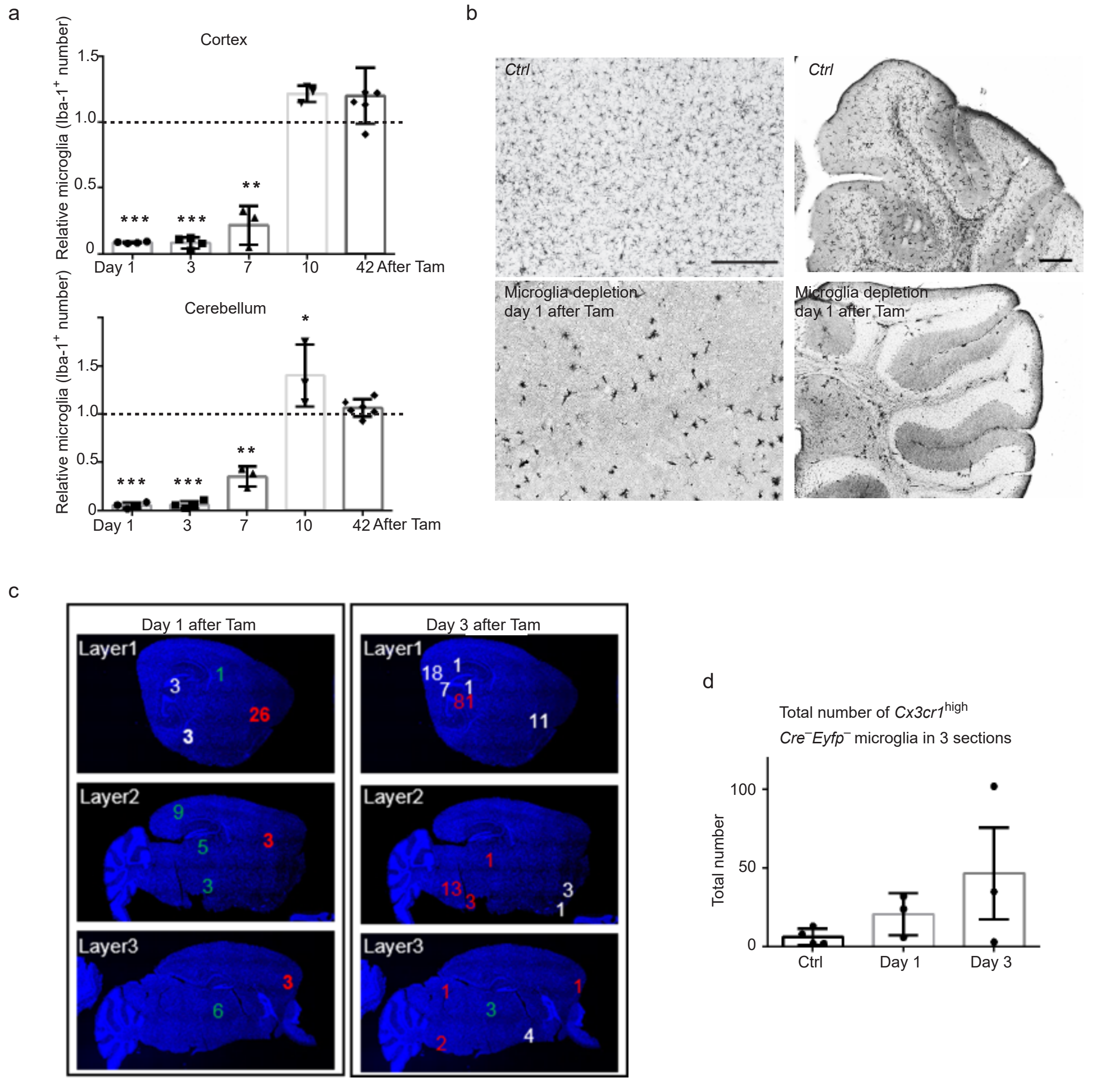

a
Cortex
Relative microglia (Iba-1+ number)
1.5
1.0
0.5
0
***
***
**
Day 1
3
7
10
42 After Tam
Cerebellum
*
Day 1
3
7
10
42 After Tam
b
Ctrl
Ctrl
Microglia depletion day 1 after Tam
Microglia depletion day 1 after Tam
c
Day 1 after Tam
Day 3 after Tam
Layer1
Layer2
Layer3
d
Total number of Cx3cr1high Cre−Eyfp− microglia in 3 sections
Total number
100
50
0
Ctrl
Day 1
Day 3

Fig. S5

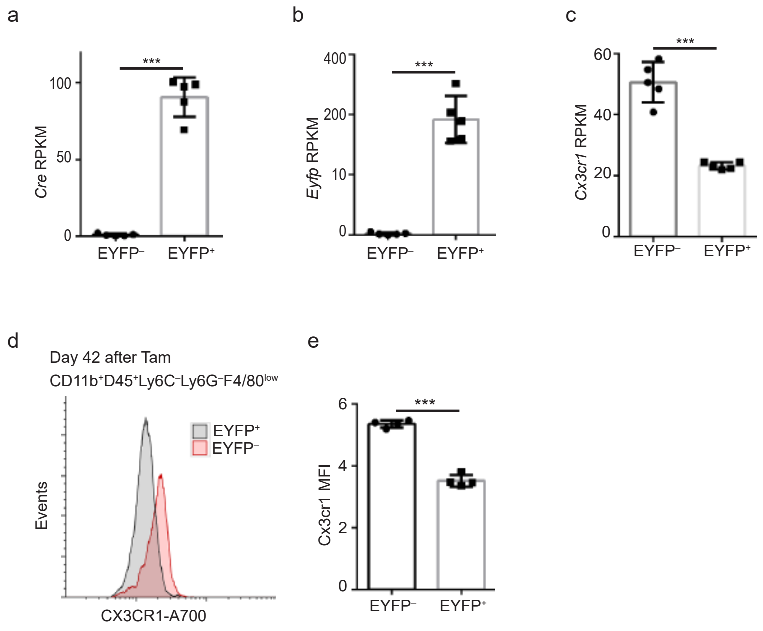
a
Cre RPKM
***
100
50
0
EYFP–
EYFP+
b
Eyfp RPKM
***
400
200
10
0
EYFP–
EYFP+
c
Cx3cr1 RPKM
***
60
40
20
0
EYFP–
EYFP+
d
Day 42 after Tam
CD11b+D45+Ly6C–Ly6G–F4/80low
EYFP+
EYFP–
Events
CX3CR1-A700
e
Cx3cr1 MFI
***
6
4
2
0
EYFP–
EYFP+

Fig. S6

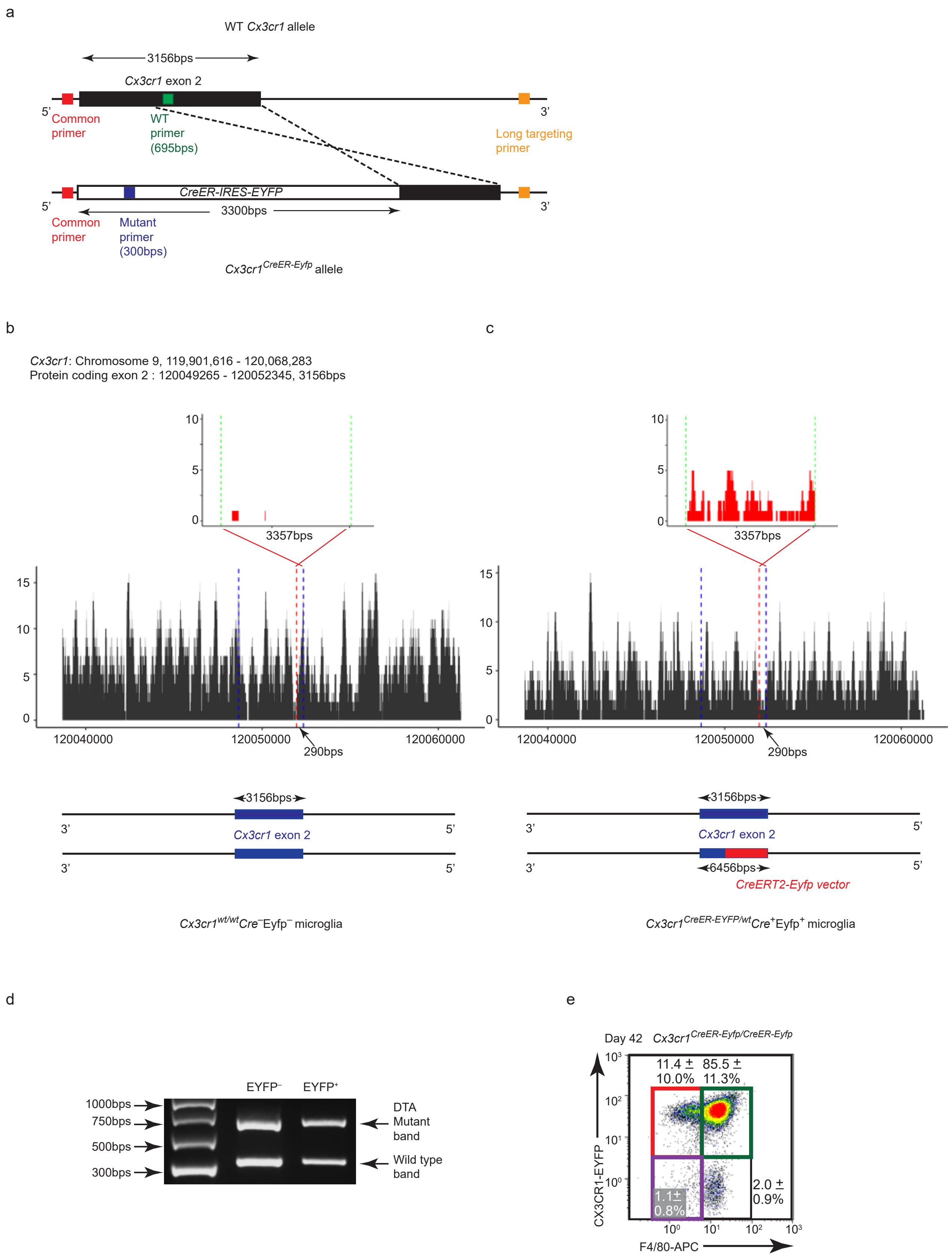
a
WT Cx3cr1 allele
3156bps
Cx3cr1 exon 2
5'
3'
Common primer
WT primer (695bps)
Long targeting primer
CreER-IRES-EYFP
3300bps
Common primer
Mutant primer (300bps)
Cx3cr1CreER-Eyfp allele
b
Cx3cr1: Chromosome 9, 119,901,616 - 120,068,283
Protein coding exon 2 : 120049265 - 120052345, 3156bps
3357bps
290bps
120040000
120050000
120060000
Cx3cr1 exon 2
Cx3cr1wt/wtCre−Eyfp− microglia
c
3357bps
290bps
3156bps
6456bps
CreERT2-Eyfp vector
Cx3cr1CreER-EYFP/wtCre+Eyfp+ microglia
d
EYFP−
EYFP+
1000bps
750bps
500bps
300bps
DTA Mutant band
Wild type band
e
Day 42 Cx3cr1CreER-Eyfp/CreER-Eyfp
11.4 ± 10.0%
85.5 ± 11.3%
1.1± 0.8%
2.0 ± 0.9%
CX3CR1-EYFP
F4/80-APC
