# Supplementary material for: An overlooked subset of Cx3cr1wt/wt microglia in the Cx3cr1CreER-Eyfp/wt mouse has a repopulation advantage over Cx3cr1CreER-Eyfp/wt microglia following microglial depletion
Source: J Neuroinflammation. 2022 Jan 21;19:20. doi: 10.1186/s12974-022-02381-6 (PMC8783445; doi:10.1186/s12974-022-02381-6)
Supplement: Supplementary file 2 — Additional file 2: Table S1. Gene list including Gene symbols, Log2 fold changes, P-values and FDR values. The table shows FDR values and P-values in ascending order. [file 12974_2022_2381_MOESM2_ESM.docx]

| **Table S1** |  |  |  |
| --- | --- | --- | --- |
| Gene | log2FC | p | FDR |
| EYFP | 13.855 | 2.37E-61 | 2.61E-57 |
| cre_bacteriophage | 10.551 | 4.00E-14 | 2.20E-10 |
| Wfdc21 | -12.824 | 1.71E-11 | 6.27E-08 |
| Camp | -13.848 | 2.54E-11 | 6.99E-08 |
| Chil3 | -13.835 | 3.47E-10 | 7.64E-07 |
| Ltf | -12.726 | 1.51E-09 | 2.7729E-06 |
| Cd177 | -12.383 | 4.03E-09 | 6.3363E-06 |
| Lcn2 | -12.917 | 5.00E-09 | 6.8643E-06 |
| Lrg1 | -12.053 | 2.50E-08 | 2.8929E-05 |
| Pglyrp1 | -12.152 | 2.71E-08 | 2.8929E-05 |
| S100a9 | -9.727 | 2.89E-08 | 2.8929E-05 |
| Hp | -10.586 | 9.60E-08 | 8.7946E-05 |
| H2-Aa | 11.794 | 1.66E-07 | 0.00014023 |
| Retnlg | -11.321 | 7.40E-07 | 0.00058083 |
| Ly6c2 | -11.259 | 8.56E-07 | 0.00062753 |
| S100a8 | -11.109 | 1.2257E-06 | 0.00084216 |
| Itgb2l | -11.057 | 1.655E-06 | 0.00107019 |
| G0s2 | -11.987 | 2.3802E-06 | 0.00145361 |
| Ngp | -9.708 | 2.7679E-06 | 0.00155956 |
| Mmp9 | -10.971 | 2.8374E-06 | 0.00155956 |
| Ly6a2 | -10.931 | 3.8787E-06 | 0.00203041 |
| Mutyh | -10.886 | 4.2721E-06 | 0.00213467 |
| Ufsp1 | -9.8 | 1.6793E-05 | 0.00802609 |
| Fbxw10 | -8.676 | 1.9365E-05 | 0.00886999 |
| Ifi47 | 9.905 | 3.2126E-05 | 0.01412654 |
| Zfp940 | 10.066 | 4.1179E-05 | 0.01741085 |
| Serpinb1a | -10.394 | 7.0544E-05 | 0.02872169 |
| Itsn1 | 10.354 | 8.5153E-05 | 0.03341354 |
| Syne1 | -9.948 | 9.0022E-05 | 0.03341354 |
| Ms4a4a | 6.134 | 9.1186E-05 | 0.03341354 |
| Mmp8 | -11.284 | 0.00012359 | 0.04382765 |
| Ralgds | -9.279 | 0.00014375 | 0.04938358 |
| Ly6g | -10.393 | 0.00017584 | 0.0585774 |
| Mmp25 | -9.172 | 0.00018605 | 0.06015305 |
| Cx3cr1 | -1.077 | 0.00019543 | 0.06138273 |
| Srf | 8.334 | 0.00025814 | 0.07882618 |
| Xlr3b | -9.117 | 0.00027549 | 0.08164371 |
| Zfp248 | -8.141 | 0.0002859 | 0.08164371 |
| Mrgpre | -11.16 | 0.00028965 | 0.08164371 |
| Lyve1 | -9.988 | 0.00033122 | 0.09102851 |
